# The NEIL glycosylases remove oxidized guanine lesions from telomeric and promoter quadruplex DNA structures

**DOI:** 10.1093/nar/gkv252

**Published:** 2015-03-26

**Authors:** Jia Zhou, Aaron M. Fleming, April M. Averill, Cynthia J. Burrows, Susan S. Wallace

**Affiliations:** 1 Department of Microbiology and Molecular Genetics, University of Vermont, Burlington, VT 05405, USA; 2 Department of Chemistry, University of Utah, Salt Lake City, UT 84112, USA

## Abstract

G-quadruplex is a four-stranded G-rich DNA structure that is highly susceptible to oxidation. Despite the important roles that G-quadruplexes play in telomere biology and gene transcription, neither the impact of guanine lesions on the stability of quadruplexes nor their repair are well understood. Here, we show that the oxidized guanine lesions 8-oxo-7,8-dihydroguanine (8-oxoG), guanidinohydantoin (Gh) and spiroiminodihydantoin (Sp) reduce the thermostability and alter the folding of telomeric quadruplexes in a location-dependent manner. Also, the NEIL1 and NEIL3 DNA glycosylases can remove hydantoin lesions but none of the glycosylases, including OGG1, are able to remove 8-oxoG from telomeric quadruplexes. Interestingly, a hydantoin lesion at the site most prone to oxidation in quadruplex DNA is not efficiently removed by NEIL1 or NEIL3. However, NEIL1, NEIL2 and NEIL3 remove hydantoins from telomeric quadruplexes formed by five TTAGGG repeats much more rapidly than the commonly studied four-repeat quadruplex structures. We also show that APE1 cleaves furan in selected positions in Na^+^-coordinated telomeric quadruplexes. In promoter G-quadruplex DNA, the NEIL glycosylases primarily remove Gh from Na^+^-coordinated antiparallel quadruplexes but not K^+^-coordinated parallel quadruplexes containing *VEGF* or *c-MYC* promoter sequences. Thus, the NEIL DNA glycosylases may be involved in both telomere maintenance and in gene regulation.

## INTRODUCTION

**Figure 1.**
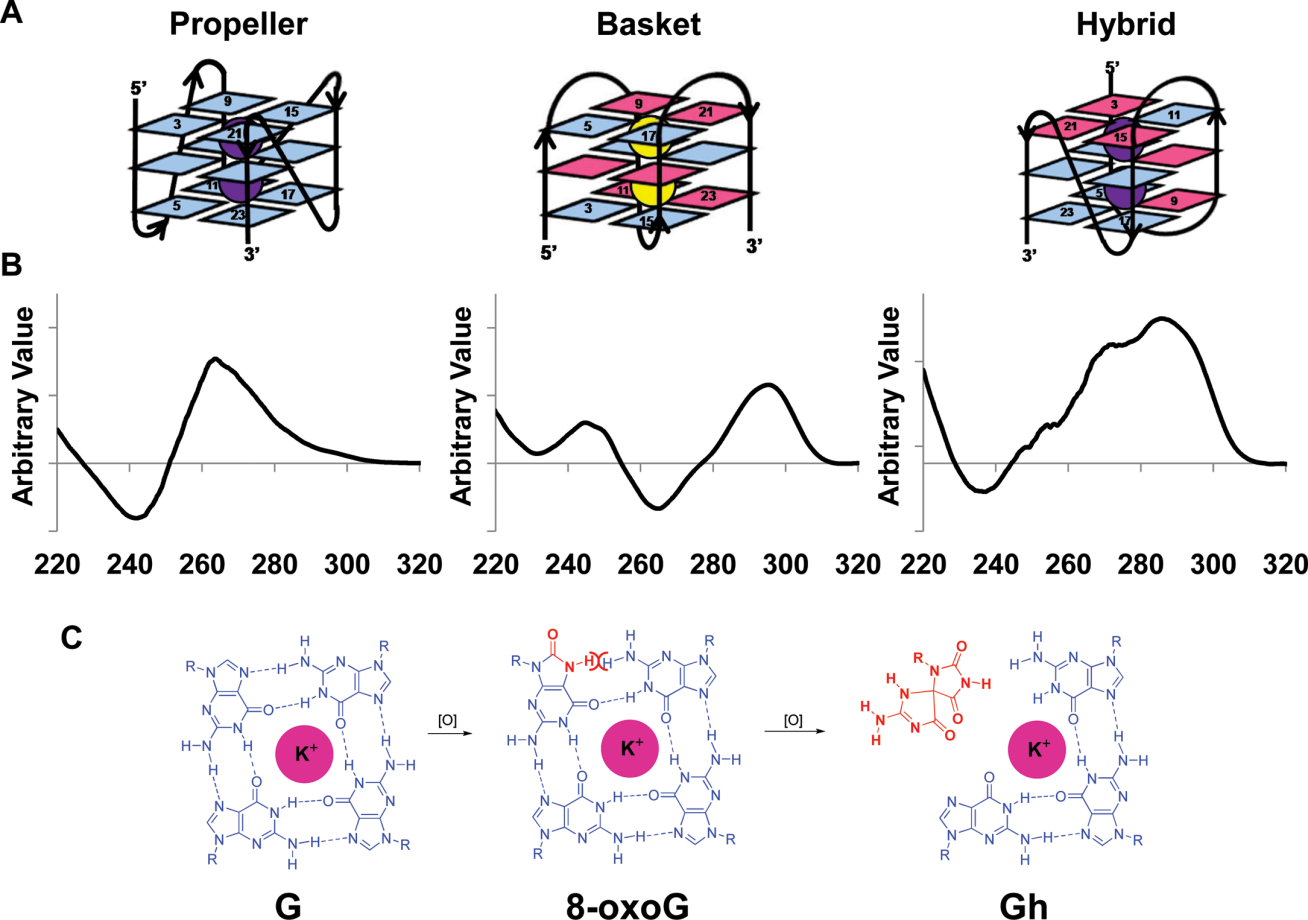
Folding, CD spectra and guanine oxidation of quadruplex DNA. (**A**) Folding of parallel propeller, antiparallel basket and hybrid (type 2) quadruplex DNA. (**B**) Representative CD spectrum of each quadruplex DNA. In a parallel quadruplex, all four strands point in one direction and the neighboring strands are connected with double reversal loops. The CD spectrum of a parallel quadruplex features a 265 nm maximum and a 240 nm minimum. The basket antiparallel quadruplex DNA has neighboring strands running in opposite directions and connected with two lateral loops and a diagonal loop. This structure features a 295 nm maximum and a 265 nm minimum. A hybrid (type 2) quadruplex has mixed strand directionalities and presents a 295 nm maximum, a 270 nm shoulder and a 235 nm minimum (see (46)). (**C**) Chemical structures of guanine (G), 8-oxoguanine (8-oxoG) and guanidinohydantoin (Gh) in the context of a G quartet.

Our cells are continuously exposed to endogenous reactive oxygen species (ROS), as well as ROS from environmental insults such as ionizing radiation. ROS damage to DNA results in strand breaks, sites of base loss as well as oxidized DNA bases (1). Both strand breaks and abasic sites can cause replication fork collapse; however, when bypass of abasic sites occurs, an adenine is preferentially inserted and can result in mutations (2). The oxidized DNA bases, if left unrepaired, may also result in mutations because of base mispairing (3,4). For example, the guanine oxidation product 8-oxo-7,8-dihydroguanine (8-oxoG) can mispair with adenine, causing G to T transversion mutations (5). 8-oxoG is susceptible to further oxidation to guanidinohydantoin (Gh) and spiroiminodihydantoin (Sp) (Figure 1C) (6,7) that are capable of mispairing with adenine and guanine (8,9). Furthermore, DNA base damages such as thymine glycol (Tg), as well as Gh and Sp, efficiently block DNA polymerases (10,11).

Base excision repair (BER) is the predominant pathway that repairs oxidative DNA base damages (4,12–16). The first enzyme in this pathway is a DNA glycosylase that recognizes and excises the damaged bases and provides substrates for the next step in the pathway. Bifunctional glycosylases also possess a lyase activity that cleaves the abasic site generated by the glycosylase activity. OGG1, NTH1, NEIL1, NEIL2 and NEIL3 are the five DNA glycosylases that are specific for removing oxidized DNA bases in human cells (1,14,17–18). OGG1 and NTH1 are housekeeping glycosylases that primarily remove oxidized purines and pyrimidines, respectively, from duplex DNA (19–21). The DNA endonuclease eight-like (NEIL) glycosylases have broader substrate specificity and are associated with particular DNA transactions. NEIL1 acts upon pyrimidine lesions such as Tg and 5-hydroxyuracil (5-OHU) in duplex DNA (22), although it also removes lesions from single-stranded and bubble DNA structures (23). Both NEIL2 and NEIL3 prefer oxidized pyrimidine and some purine damages in single-stranded DNA (23,24), and we have previously shown that mouse Neil3 removes lesions from quadruplex DNA (25). Although, 8-oxoG is not a substrate for any of the NEIL glycosylases, its further oxidation products, Gh and Sp, are the best substrates for all three enzymes (24,26–28). In terms of cellular function, NEIL1 acts in concert with the replication fork removing lesions before they are encountered by the replicative DNA polymerases (29) while NEIL2 appears to function during transcription-coupled repair (23,30). The cellular function of NEIL3 remains elusive. However, expression of Neil3 is restricted to highly proliferating cells, including embryonic stem cells, pluripotent cells in brain and hematopoietic cells in mice (31,32) and cancer cells in human (31,33).

G-quadruplex (G4) is a four-stranded DNA structure containing two or more layers of guanine quartets. In each layer, four guanines Hoogsteen base pair to each other. Monovalent cations (i.e. K^+^ and Na^+^) stabilize G4 DNA by coordinating layers of guanine quartets. Quadruplex structures have been proposed to play regulatory roles during lagging strand replication, gene transcription, mRNA translation and telomeric DNA elongation (34). Bioinformatics studies revealed that G4-forming sequences are prevalent throughout the human genome. Interestingly, the distribution of these sequences is not random and telomere regions and promoter regions of genes are the two places enriched for potential G4-forming sequences (35,36).

Telomeres are DNA–protein complexes at the ends of chromosomes that prevent degradation, undesired fusion and improper activation of DNA damage response pathways (37,38). Human telomeres consist of 2–20 kb of 5′-TTAGGG-3′ repeats, the bulk of which is double-stranded with an ∼150 bp 3′ single-stranded overhang at the very end (39,40). Telomeric DNA sequences can form G4 structures under physiological salt conditions *in vitro* (41,42). A number of quadruplex structures containing the human telomeric sequence have been solved in both Na^+^ (43) and K^+^ (44,45) solutions using nuclear magnetic resonance (NMR) and X-ray crystallography. Circular dichroism (CD) has been used as a routine method to study G-quadruplex DNA folding. There are three common quadruplex topologies based on the directionality of neighboring strands, namely parallel, antiparallel and hybrid (type 1 and type 2), each having a characteristic CD spectrum (Figure 1A and B) (46). Although most structural studies of quadruplexes have been done *in vitro*, there is increasing evidence supporting the existence of quadruplex structures in cells (47,48).

Because guanine has the lowest redox potential among the four bases, telomere DNA provides a vulnerable target for oxidation both *in vitro* (49,50) and in cells (51). Accumulation of base damages such as 8-oxoG in telomeres may hinder telomerase activity (52) and disrupt the telomere-guarding shelterin complex (53). Thus, base damages in telomeres have to be repaired in order to preserve telomere integrity. Shortening and loss of function of telomeres can also lead to cell senescence and premature aging syndromes in humans (54). On the other hand, telomere lengthening due to overactive telomerase has been linked to cancer (55).

Two groups have shown that promoter regions of many genes are rich in G4-forming sequences (35,36). Over 40% of human genes have at least one potential G4-forming sequence near their promoter regions (56). Quadruplex DNA structures present at promoter sites have been linked to the transcription of downstream genes. For example, the nuclease hypersensitive element III1 (NHE III1) upstream of the P1 promoter of the human proto-oncogene *c-MYC* controls up to 90% of the total *c-MYC* transcription (57,58), which was later demonstrated to involve quadruplex formation at this site (59). The human vascular endothelial growth factor (VEGF) is a key regulator of angiogenesis and plays an important role in tumor survival, growth and metastasis (60,61). The −88 to −50 bp region relative to the transcription initiation site of the *VEGF* gene, which contains multiple transcription factor binding sites (i.e. Sp1 binding sites (62)), forms a quadruplex structure and has been shown to function in *VEGF* transcription (63,64). Furthermore, quadruplex-interactive agents have been shown to repress both *VEGF* and *c-MYC* expression in human tumor cells (65,66). G-quadruplexes in promoter regions of genes have emerged as therapeutic targets in oncology, and stabilization of such structures has potential as a novel anticancer strategy (67).

We have previously shown that mouse Neil3 and human NEIL1 glycosylases are able to remove damaged bases from antiparallel G4 DNA formed in Na^+^ solution (25). Here, we studied the impact of hydantoin lesions on the thermostability of telomeric G4 DNA formed in K^+^ solution as well as the removal of hydantoins from such structures. We have also investigated the removal of hydantoin lesions from quadruplexes formed by promoter sequences, using *VEGF* and *c-MYC* promoter sequences as models.

## MATERIALS AND METHODS

### Oligodeoxyribonucleotide synthesis and purification

Sequences of oligodeoxyribonucleotides (ODNs) used in this study are listed in Table 1. All 8-oxoG-containing ODNs were synthesized and deprotected by the DNA-peptide core facility at the University of Utah following the manufacturer's protocols (Glen Research, Sterling, VA, USA). The crude samples were purified by semi-preparative ion-exchange high performance liquid chromatography (HPLC) and desalted by dialysis against ddH_2_O. The purified 8-oxoG ODNs were used in the synthesis of Sp and Gh. Synthesis of Gh and Sp was achieved by oxidation using K_2_IrBr_6­­_ in solution. Samples were purified by analytical ion-exchange HPLC and desalted by dialysis against ddH_2_O. The detailed protocols for synthesis and purification of Gh and Sp can be found in Supplemental Information. Product purity was determined by analytical ion-exchange HPLC and product identity was determined by electrospray ionization - mass spectrometry (ESI-MS) (Supplementary Figures S1 and S2). Other ODNs used in this study, including the furan-containing ODNs, were purchased from Midland Certified Reagent Co. (Midland, TX, USA), and were gel purified. All ODNs were quantified by NanoDrop spectrophotometry using their extinction coefficients.

**Table 1.** ODN sequences used in this study

Name	Sequence	Description
Tel-9	5′-TAGGGTTAXGGTTAGGGTTAGGGTT-3′	X = OG, Gh, (*S*)-Sp or (*R*)-Sp at 5′
Tel-10	5′-TAGGGTTAGXGTTAGGGTTAGGGTT-3′	X = OG, Gh, (*S*)-Sp or (*R*)-Sp in the middle
Tel-11	5′-TAGGGTTAGGXTTAGGGTTAGGGTT-3′	X = OG, Gh, (*S*)-Sp or (*R*)-Sp at 3′
5-repeat-5′	5′-TAXGGTTAGGGTTAGGG-TTAGGGTTAGGGTT-3′	X = OG or Gh at the 5′ repeat of the 5-repeat sequence
5-repeat-mid	5′-TAGGGTTAGGGTTAXGG-TTAGGGTTAGGGTT-3′	X = OG or Gh at middle repeat of the 5-repeat sequence
5-repeat-3′	5′-TAGGGTTAGGGTTAGGG-TTAGGGTTAXGGTT-3′	X = OG or Gh at the 3′ repeat of the 5-repeat sequence
F9	5′-TAGGGTTAFGGTTAGGGTTAGGGTT-3′	Furan at 5′ of GGG
F10	5′-TAGGGTTAGFGTTAGGGTTAGGGTT-3′	Furan at the middle of GGG
F11	5′-TAGGGTTAGGFTTAGGGTTAGGGTT-3′	Furan at 3′ of GGG
F13	5′-TAGGGTTAGGGTFAGGGTTAGGGTT-3′	Furan in a loop region
*VEGF*-12	5′-CGGGGCGGGCCXGGGGCGGGGT-3′	X = OG or Gh
*VEGF*-14	5′-CGGGGCGGGCCGGXGGCGGGGT-3′	X = OG or Gh
*c-MYC*-8	5′-TGAGGGTXGGGAGGGTGGGGAA-3′	X = OG or Gh
*c-MYC*-11	5′-TGAGGGTGGGXAGGGTGGGGAA-3′	X = OG or Gh
R25	5′-ATTGACTTCTCCACTTGCTATTGAC-3′	A random 25-mer used as a ssDNA control
R-Gh	5′TGTTCATCATGCGTC[Gh]TCGG-TATATCCCAT-3′	Control sequence with Gh
R-8-oxoG	5′-TGTCAATAGCAAG[8-oxoG]GGAGAA-GTCAATCGTGAGTCT-3′	Control sequence with 8-oxoG
R-Tg	5′-TGTCAATAGCAAG[Tg]GGAGAA-GTCAATCGTGAGTCT-3′	Control sequence with Tg

Tel, telomere DNA sequence with four repeats; 5-repeat, telomere DNA sequence with five repeats.

### CD analysis

G-quadruplex folding was characterized by CD spectroscopy using a JASCO spectrometer. First the G-quadruplex samples were annealed at a 10 μM concentration in 20 mM HEPES (4-(2-hydroxyethyl)-1-piperazineethanesulfonic acid) buffer (pH 7.4) and 100 mM KCl (or NaCl) by heating the samples at 90°C for 5 min followed by slowly cooling them down to room temperature, after which they were placed at 4°C for 48 h prior to analysis. CD analysis of each sample was achieved on the annealed G-quadruplexes by scanning and averaging 10 scans of the CD spectrum. The data were background subtracted, normalized and plotted as molar ellipticity versus wavelength.

### T_m_ analysis

Thermal melting studies were conducted on the annealed G-quadruplex samples. The CD samples were diluted to a 3 μM G-quadruplex concentration using the buffer system described above. Next, the samples were placed in T_m_ cuvettes that were put into a UV–vis spectrometer (Shimadzu) equipped with a temperature control unit. The samples were thermally equilibrated at 20°C for 5 min prior to commencing the heating cycle. The samples were heated at a rate of 0.5°C/min and thermally equilibrated after each step for 1 min to achieve a final temperature of 100°C that was then reversed back to room temperature via an identical decreasing temperature method. Absorbance readings at 260 and 295 nm were taken every 1°C. The data were plotted as absorbance at 295 nm versus temperature and the T_m_ values were determined from the first derivative of the curve as analyzed by the instrument's software (Shimadzu). The average and standard deviation values of quadruplicate trials are reported.

### Enzyme purification

Cloning, expression and purification of the glycosylase domain of the human NEIL3 glycosylase were recently published (68). Unless otherwise specified, NEIL3 used in this study was the glycosylase domain (NEIL3-GD) of the enzyme. NEIL1 and NTH1 glycosylases were purified as previously described (69). NEIL2, OGG1 and APE1 were from our laboratory stocks and were purified as previously described (70). Because the cation identity is critical for folding of the quadruplex DNA, enzymes prepared in Na^+^-containing buffer were dialyzed against K^+^-containing buffer when assaying K^+^-coordinated quadruplexes. Protein concentrations were determined by the BCA Protein Assay Kit (Thermo Scientific Pierce). The percentage of active glycosylase was determined by the Schiff base assay (for NEIL1, NEIL2, NEIL3 and OGG1) (70) or by the molecular accessibility method (for NTH1) (71), and all protein concentrations reported in this study were corrected for the active enzyme percentage.

### DNA substrate preparation

Lesion-containing ODNs were ^32^P-labeled at the 5′ end by T4 polynucleotide kinase (NEB). The labeled ODNs were ethanol precipitated as previously described (72). Substrates typically contained one part hot ODN and nine parts cold ODN. All G4-forming ODNs were annealed in quadruplex folding buffer (Qu buffer), which contains 20 mM HEPES-KOH (pH 7.4), 100 mM KCl and 1 mM EDTA. HEPES-NaOH (pH 7.4) and 100 mM NaCl were used when making Na^+^-coordinated quadruplex DNA. To fold quadruplex DNA, the ODN in Qu buffer was heated at 95°C for 5 min and slowly cooled to room temperature. The mixture was then stored at 4°C overnight before being used for assays.

### Native gel electrophoresis

G4 DNA substrates were prepared as described above. Samples were loaded with 5% glycerol on to a 16% acrylamide (29:1) native gel with 0.5× Tris-Borate-EDTA (TBE) and 100 mM KCl. The native gel was run in 0.5× TBE plus 100 mM KCl at 3 volts/cm overnight in 4°C, and the gel was then dried and exposed to a phosphorimager screen for detection.

### Glycosylase/lyase activity assays

Glycosylase assays were done in the quadruplex reaction buffer, which contains 20 mM HEPES-KOH pH 7.4, 100 mM KCl, 1 mM EDTA, 0.1 mg/ml BSA and 1 mM DTT. In the case of Na^+^-coordinated quadruplexes, HEPES-NaOH and NaCl were used in the buffer. Substrate concentration was typically 10 nM unless otherwise specified. Enzymes and substrates were incubated at room temperature or 37°C as indicated. To measure glycosylase plus lyase activities, reactions were quenched by adding an equal volume of FE buffer (96% formamide, 20 mM EDTA, 0.1% bromophenol blue and 0.1% xylene cyanol) directly. To measure only the glycosylase activity, reactions were terminated by adding NaOH to a final concentration of 0.33 N and heated at 95°C for 4 min. An equal volume of FE buffer was added to the reactions before loading on to a 12% urea gel for separation. The gel was dried and exposed on a phosphorimager screen. Finally, bands from the screen were scanned by Molecular Imager PharosFX Plus (Bio-Rad Laboratories) and quantified by Quantity One software (Bio-Rad Laboratories).

### Single-turnover kinetics

For single-turnover kinetics analysis of NEIL3-GD, 10 nM of substrate was incubated with 100 nM of enzyme in a total volume of 150 μl. An aliquot of 10 μl reaction mixture was taken out and quenched with 5 μl of 1 N NaOH. The same procedure was then carried out to obtain the gel images. The intensity of the bands was quantified by Quantity One and analyzed by GraphPad Prism 6 software. The catalytic rate (*k*_obs_) was determined by fitting the data to a one phase association model.

## RESULTS

### Guanine oxidation alters the folding of telomeric quadruplexes and reduces their thermostability in KCl solution

**Figure 2.**
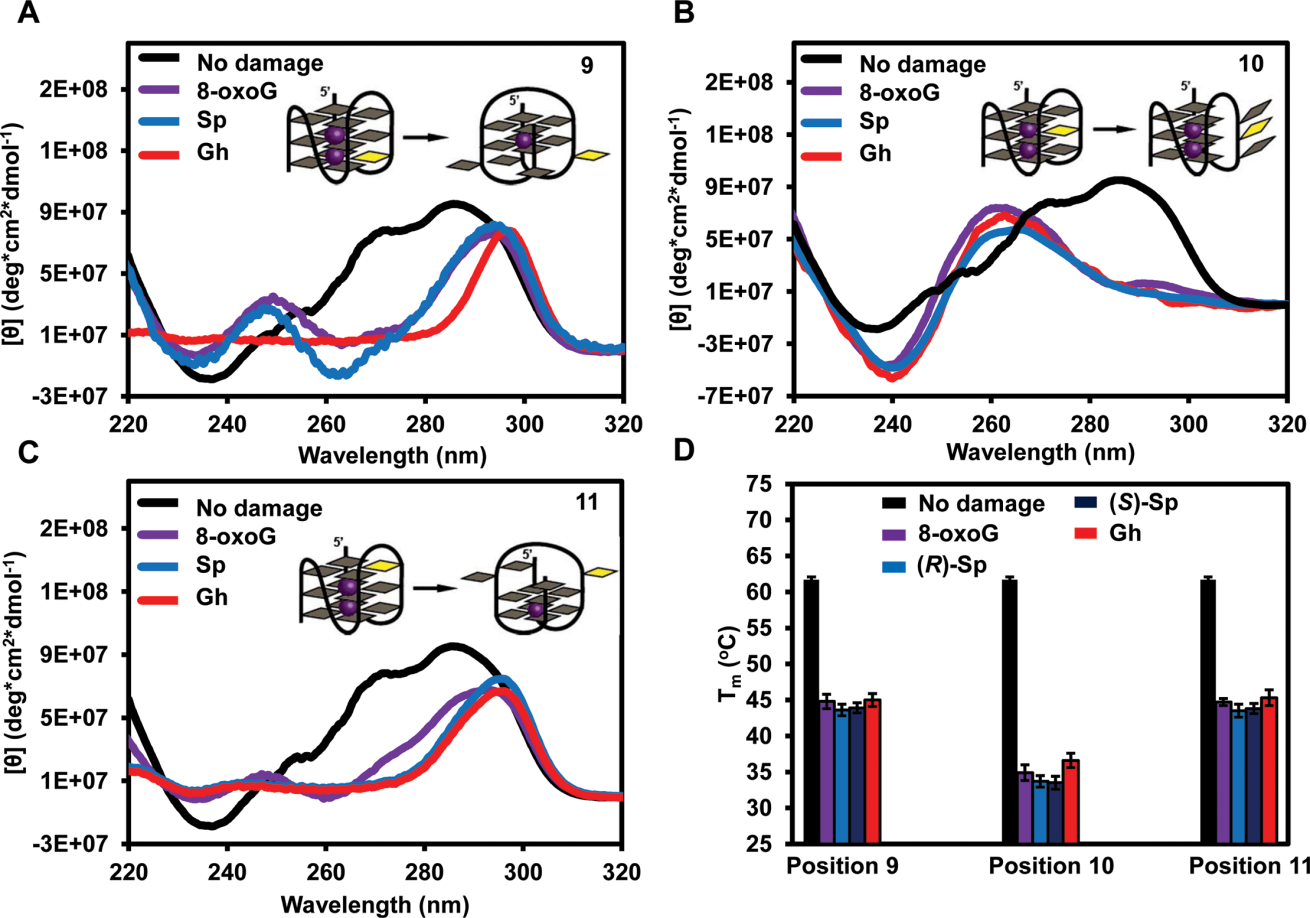
Guanine oxidation alters quadruplex folding and reduces the thermostability of quadruplexes. All samples were annealed in quadruplex folding buffer containing K^+^. CD spectra were recorded at 20°C. (**A**) The CD spectra of telomeric DNA with an 8-oxoG, Gh or Sp lesion at the 5′ position of a GGG triad (position 9). (**B**) The CD spectra of telomeric DNA with a guanine lesion in the middle of a GGG triad (position 10). (**C**) The CD spectra of telomeric DNA with a guanine lesion 3′ of a GGG triad (position 11). CD spectra for the Sp-containing quadruplexes were obtained on a mixture of diastereomers. (**D**) Guanine lesions reduce the thermostability of telomeric quadruplex DNA. Mean and standard deviation of the T_m_ values calculated from four experiments are shown.

All telomeric DNA sequences were folded in the quadruplex folding buffer containing K^+^, in which the ODN without a lesion folds into a hybrid quadruplex structure, as previously determined (73,74). The CD spectrum of each lesion-containing telomeric DNA was recorded in order to reveal the structure under the assay conditions. First, telomeric G4 DNA with a guanine lesion in an exterior quartet (positions 9 and 11) gave similar CD spectra. These folds have maxima at 295 and 245 nm, and a minimum around 265 nm (Figure 2A and C), which are consistent with an antiparallel fold that adopts a basket-like topology (75). In addition, the thermal melting (T_m_) studies for the G4 DNAs with lesions at either exterior quartet gave significantly reduced T_m_ values (−17°C) for 8-oxoG, Gh and the two Sp diastereomers (Figure 2D). Based on the CD spectra, the lesions at positions 9 and 11 convert the fold from a hybrid to a basket under identical conditions with a significant reduction in thermal stability. Furthermore, 8-oxoG, Gh and the Sp diastereomers are not capable of Hoogsteen H-bonding in the guanine quartet (Figure 1C); therefore, we propose that the damage induces the basket topology with two quartets and the lesion residing in a poorly defined context (Figure 2A and C).

In contrast, the ODN with a guanine lesion in the center quartet (position 10) showed a dramatic change in the CD spectra recorded compared to the native hybrid fold (Figure 2B). Placement of damage in the center quartet gave a maximum peak at 263 nm and a minimum at 239 nm. The T_m_ values for all lesions in the middle quartet were ∼30°C lower than the native hybrid fold (Figure 2D). First, these observations were anticipated because 8-oxoG, Gh and the Sp diastereomers cannot Hoogsteen base pair, and Gh and Sp are not planar, and therefore, cannot π-stack in the middle of a G4 fold (Figure 1C). Based on these observations, we propose that the damage is everted and the remaining Gs fold to a triplex-like structure with limited stability (Figure 2B). Consistent with this hypothesis are the CD profiles for triplex structures that were further characterized by optical tweezers (76) and nanopore measurements (77). Taken together, introduction of lesions of G oxidation causes the human telomere G4 to adopt significantly different topologies than the non-damage-containing sequence with significant reduction in their thermal stability. Analogous results have been reported for 8-oxoG in a similar sequence (78).

**Figure 3.**
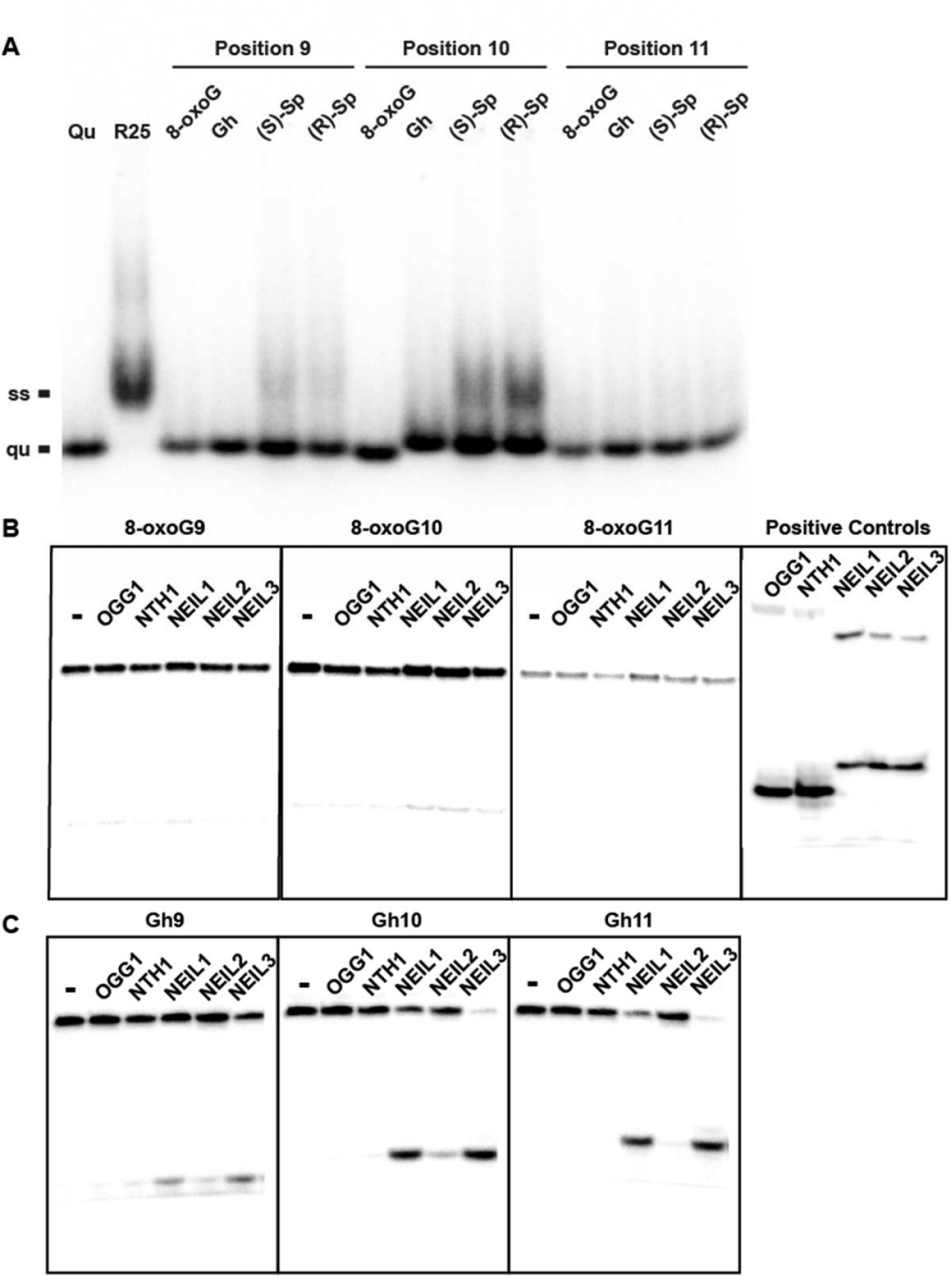
Hydantoin lesions but not 8-oxoguanine are readily removed by glycosylases from quadruplex/triplex DNA. (**A**) Native gel electrophoresis shows that guanine lesion-containing telomere ODNs (Tel sequences) form compacted secondary structures and migrate faster than single-stranded DNA. Qu, quadruplex structure formed by Tel sequence without a lesion. R25, a single-stranded DNA control with random sequence. (**B**) No glycosylase shows activity on 8-oxoG-containing quadruple/triplex DNA. In the positive control, duplex R-8-oxoG was used for OGG1, duplex R-Tg was used for NTH1 and single-stranded R-Gh was used for NEIL1, 2 and 3. (**C**) NEIL1 and NEIL3 but not NEIL2 remove Gh from quadruplex DNA. Ten nanomolar of each substrate was incubated with 200 nM of each glycosylase at room temperature for 30 min, and the reaction was stopped by adding NaOH and heating.

Next, the compacted secondary structures of these guanine lesion-containing G4 DNAs were assayed on a native gel (Figure 3A). Telomeric DNA with a guanine lesion in an exterior quartet (position 9 or position 11) migrated faster than the single-strand random sequence (R) control and formed uniform bands, which suggested that these sequences form compact DNA structures, presumably G-quadruplexes. Telomeric DNA with a Gh, (*S*)-Sp or (*R*)-Sp in a middle quartet (position 10) migrated a little more slowly than the quadruplexes, agreeing with the prediction of a triplex DNA structure for these sequences. Additionally, a minor band was observed that appears to be single-stranded DNA in (*S*)-Sp-10 and (*R*)-Sp-10; this observation is consistent with the greatly reduced stability of a quadruplex when a damaged base is introduced into the middle quartet (79).

### Hydantoin guanine lesions but not 8-oxoguanine are readily removed by glycosylases NEIL1 and NEIL3 from telomeric quadruplex DNA and triplex DNA

Previously, we demonstrated that OGG1, NTHL1, NEIL1, NEIL2 and NEIL3 DNA glycosylases are incapable of removing 8-oxoguanine from an antiparallel basket G4 folded in NaCl solution (25). In the current study, repair of 8-oxoguanine in human telomere G4 was studied in KCl solution, which is more similar to cellular conditions. Similar to the Na^+^ study, none of the DNA glycosylases, including OGG1, initiated repair of 8-oxoG in any of the three positions studied (9, 10 and 11) (Figure 3B). Note that these reactions were carried out with 20-fold excess of enzyme relative to substrate.

On the other hand, Gh in the quadruplex/triplex structure can be rapidly removed by human DNA glycosylases NEIL1 and NEIL3 (Figure 3C). Similar results were observed for (*S*)-Sp and (*R*)-Sp in the quadruplex/triplex structure (Supplementary Figure S2). However, NEIL2 and NTH1 did not excise any of the hydantoin lesions from quadruplex or triplex DNA; this result is in contrast to the hydantoins being good substrates for NEIL2 in single-stranded DNA (27) and moderate substrates for Nth in duplex DNA (80).

We also tested the glycosylase plus lyase activities of the NEIL glycosylases. Telomeric quadruplex DNA with Gh was used as substrate, and the reactions were stopped by formamide/EDTA buffer to visualize the lyase activity after base removal. NEIL1 and NEIL3 were able to hydrolyze abasic sites, primarily at positions 10 and 11 (Supplementary Figure S3). However, the lyase activity of NEIL3 was low including the control ssDNA substrate, which is consistent with observations that mouse (68) and human NEIL3 (81) are primarily monofunctional glycosylases. In addition, NEIL2 showed no glycosylase/lyase product as predicted, since NEIL2 does not have glycosylase activity on Gh in quadruplex DNA in the first place (Supplementary Figure S3B and C).

### Lesions at the preferred oxidation site in telomeric quadruplex DNA are resistant to excision by NEIL1 and NEIL3

**Figure 4.**
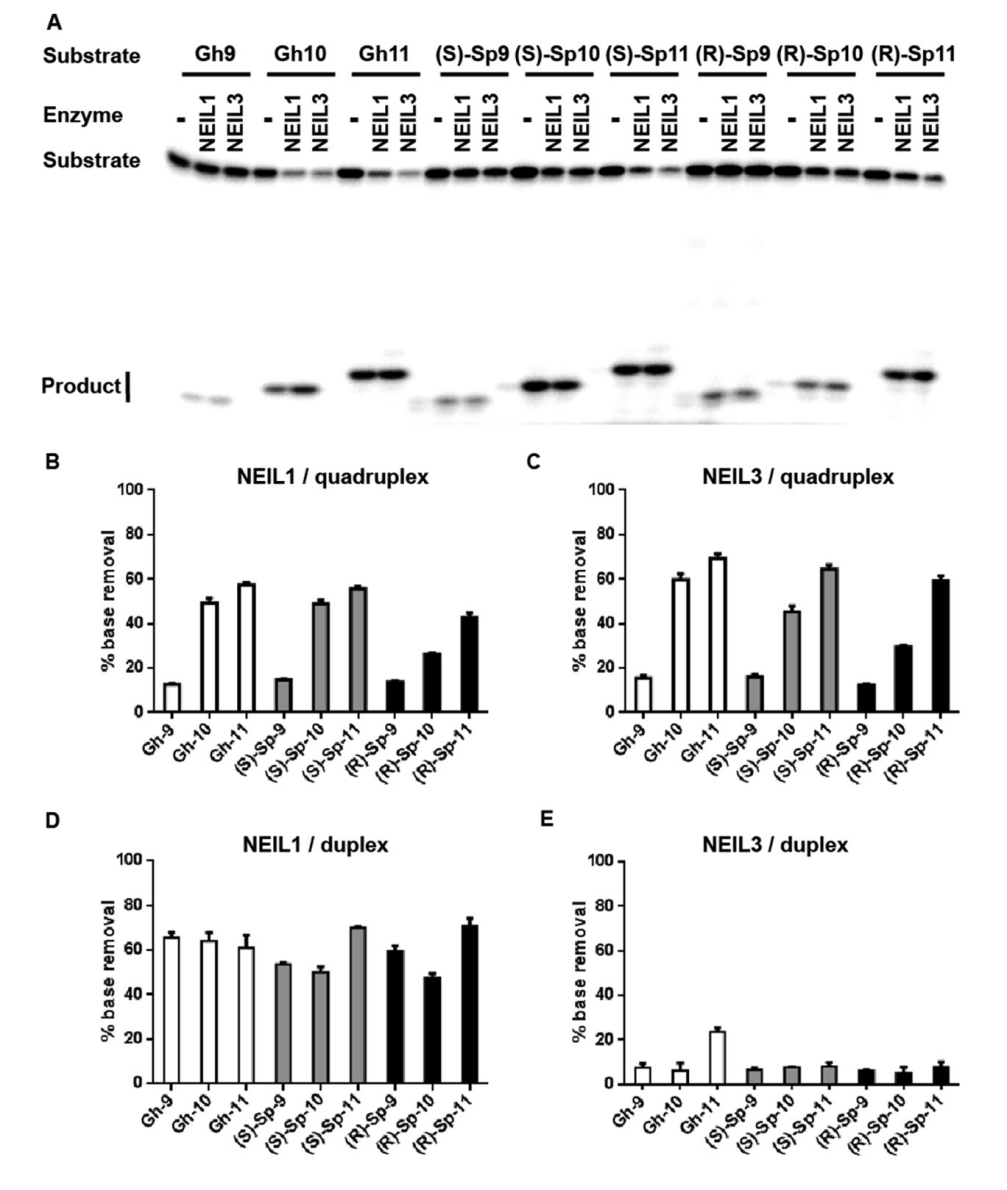
Lesions at the preferred oxidation site in quadruplex DNA are resistant to excision by glycosylases. (**A**) A representative gel image of the glycosylase assays. (**B, C**) Quantification of the glycosylase activities of NEIL1 (B) and NEIL3 (C) on quadruplex/triplex DNA containing Gh, (*S*)-Sp and (*R*)-Sp at three positions. (**D, E**) Quantification of the glycosylase activities of NEIL1 (D) and NEIL3 (E) on the corresponding duplex substrates. Average percentage of base removal and the standard deviation from three replicates are shown. All reactions contained 10 nM substrate and 4 nM active enzyme. All reactions were stopped by adding NaOH and heating after incubation at room temperature for 30 min.

Our previous study on quadruplex oxidation revealed that hydantoins were the major oxidation products, and identified the 5′ guanine of a GGG stretch as the most reactive site toward one-electron oxidants in the G4 context (50). With this information, the glycosylase repair efficiency toward G lesions was measured for the hydantoin lesions specifically synthesized at each site of the G_9_G_10_G_11_ run. We first measured the glycosylase activities of NEIL1 and NEIl3 on these substrates under multiple turnover conditions (Figure 4). These results show that NEIL1 and NEIL3 preferentially remove hydantoins from the middle and 3′-sites of a GGG stretch (position 10 and position 11), while damages at the 5′-site of a GGG (position 9) were not efficiently removed (Figure 4A–C). Specifically, when 10 nM hydantoin (Gh, (*S*)-Sp or (*R*)-Sp)-containing G4 DNA was incubated with 4 nM enzyme for 30 min, about 50% of the lesions located at the middle or 3′ sites were removed, while only about 15% of the 5′ lesions were removed. These observations also support the conclusion that it is the position of the hydantoin in the G4 DNA, and not its chemical architecture, that dominates the removal efficiency (Figure 4A–C).

We have previously shown that the sequence surrounding the lesion may significantly affect the glycosylase activity (sequence context effect) (25). To determine if the reason why lesions at position 9 were inefficiently removed by NEIL1 and NEIL3 was due to sequence context effect, double-stranded substrates with the same sequences were assayed under the same condition. Figure 4D shows that NEIL1 removes Gh, (*S*)-Sp and (*R*)-Sp at the three positions with the same or comparable efficiency. As expected, NEIL3 exhibits a lower efficiency than NEIL1 (Figure 4E) on duplex DNA; however, there was no observable sequence context effect on the ability of NEIL3 to remove the Sp diastereomers. Although NEIL3 had higher activity on dsGh11 than dsGh9, dsGh9 was a better substrate than dsGh10, which does not explain the inefficient lesion removal from position 9 in the quadruplex. Comparison of the G4 and duplex results suggests that the differential activity of NEIL1 and NEIL3 at positions 9, 10, 11 in G4 is not a result of sequence context, but rather the difference in the structures of the lesion-bearing G4 DNAs.

**Figure 5.**
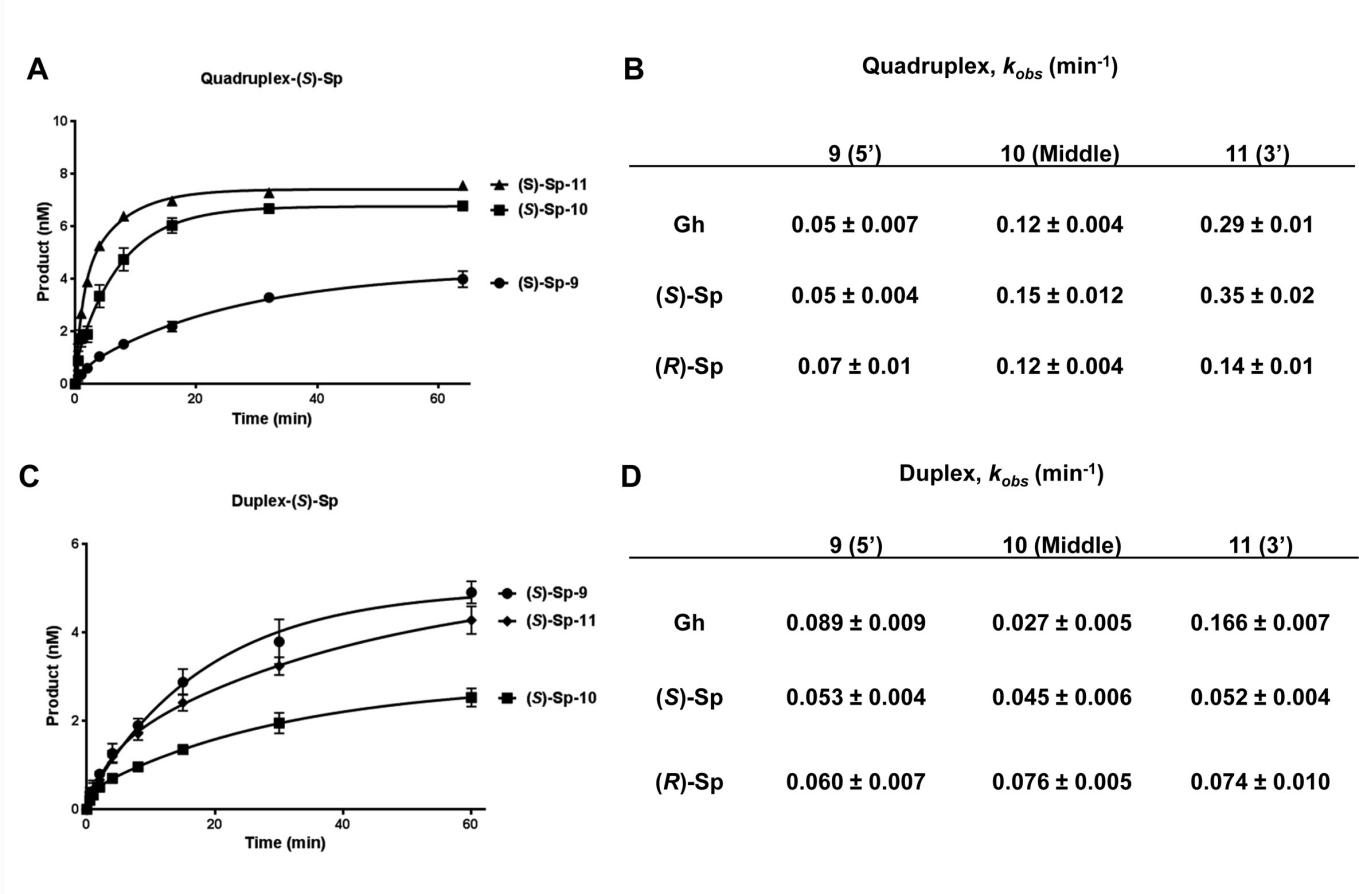
Catalytic rates (*k*_obs_) of NEIL3 glycosylase on quadruplex DNA confirms poor cleavage of the 5′ lesion. (**A**) Representative time courses of NEIL3 on (*S*)-Sp-containing quadruplex/triplex DNA used for calculating *k*_obs_. (**B**) Catalytic rates of NEIL3 on three hydantoin lesions at three different positions in quadruplex/triplex DNA. (**C**) Representative time courses of NEIL3 on (*S*)-Sp-containing duplex DNA used for calculating *k*_obs_. (**D**) Catalytic rates of NEIL3 on three hydantoin lesions at three different positions in duplex DNA. All reactions contained 10 nM substrate and 100 nM active NEIL3 enzyme. Reactions were stopped by mixing with NaOH and heating after incubating at room temperature at the indicated time points.

In order to better compare the base removal efficiency, kinetic rates of NEIL3 were measured under the single-turnover conditions (enzyme in a 10-fold excess) (Figure 5). As observed above, the 5′ hydantoin lesions were removed from the quadruple/triplex at the slowest rate by NEIL3 (Figure 5A and B). For all three hydantoins, the time course studies revealed that NEIL3 removes the middle and 3′ hydantoins much more rapidly (Figure 5A for (*S*)-Sp and Supplementary Figure S4 for (*R*)-Sp and Gh). Not surprisingly, the kinetic rates (*k*_obs_) again showed that NEIL3 preferred the middle and 3′ hydantoins over those in the 5′ position, with differences ranging from 2- to 7-fold (Figure 5B). We also measured *k*_obs_ for NEIL3 using the corresponding duplex DNA under the same conditions (Figure 5C and D). There was no significant difference in the removal of the ds(*S*)-Sp and ds(*R*)-Sp by NEIL3 among three positions studied, and the differences among dsGh9, dsGh10 and dsGh11 do not explain the inefficiency of removal of Gh9 in the quadruplex DNA context (Figure 5C and D). Therefore, we conclude that hydantoin lesions present in the major site for damaging guanine (5′, position 9) are not efficiently removed by NEIL1 or NEIL3 glycosylases, and that the structural differences of G4 DNA, but not the sequence context effect or lesion identity of the hydantoin, play the dominant role in the efficiency of damage removal from the quadruplexes.

### An extra telomeric 5′-TTAGGG-3′ repeat allows alternative folding of the quadruplex and facilitates removal of the resistant 5′ hydantoin by the NEIL glycosylases

**Figure 6.**
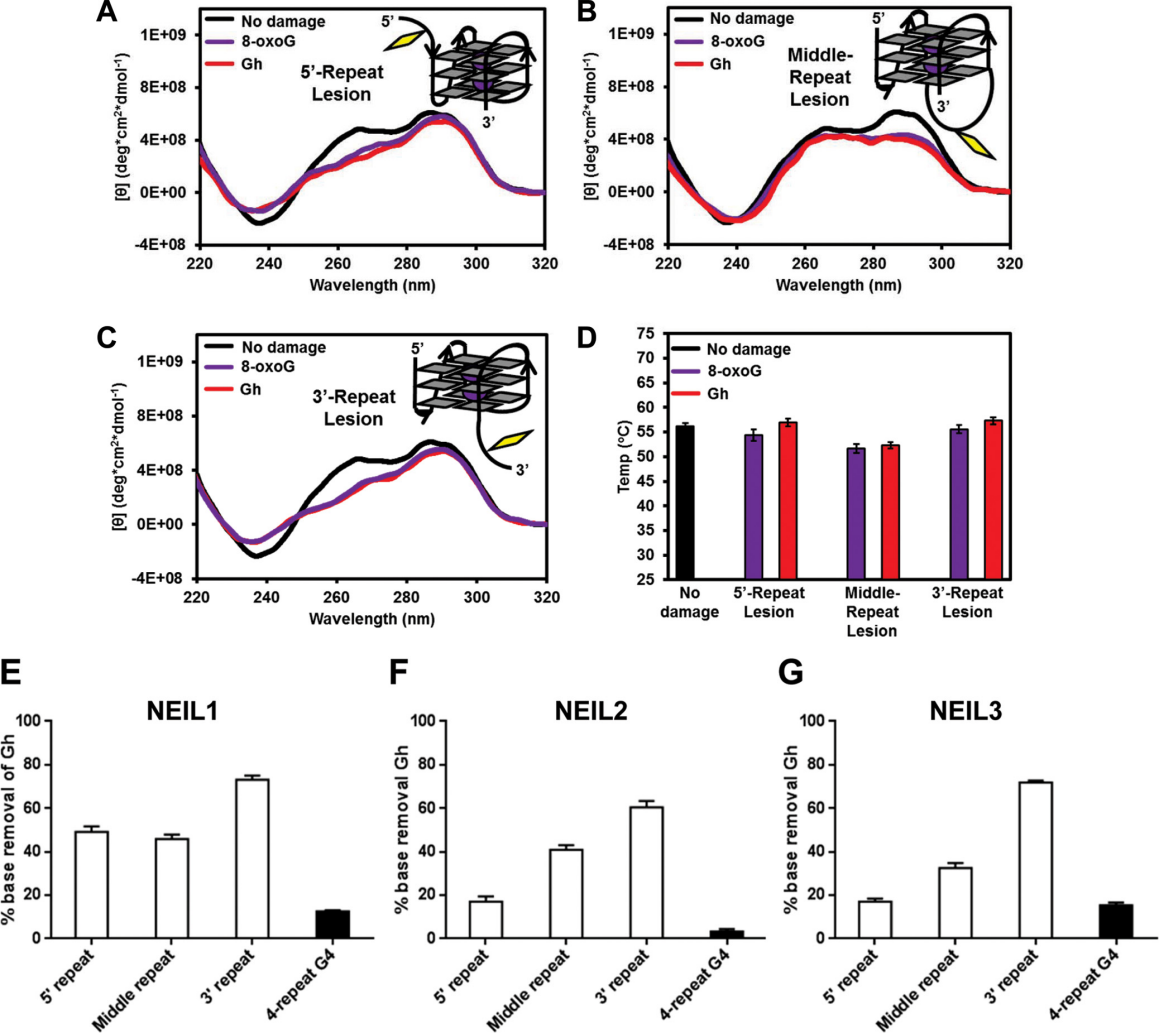
Alternative folding of the quadruplex facilitates removal of the resistant 5′ hydantoin. (**A–C**) CD spectra of folded 5-repeat telomeric sequences with 8-oxoG or Gh at the 5′, middle or 3′ TTAGGG repeat. (**D**) Melting temperature (T_m_) of 5-repeat quadruplexes with 8-oxoG and Gh at the 5′, middle or 3′ TTAGGG repeat. (**E–G**) Quantification of Gh removal in 5-repeat quadruplexes by NEIL1 (E), NEIL2 (F) and NEIL3 (G). The 5-repeat quantification results were compared to the values obtained with 4-repeat quadruplexes with Gh at position 9 (5′ of GGG triad). Ten nanomolar of substrate and 4 nM of active enzyme were incubated at room temperature for 30 min.

The high reactivity of the 5′ G toward oxidation leading to hydantoin products that are not properly removed by the BER glycosylases created a paradox. Therefore, we looked at a more relevant telomere context by adding a fifth 5′-TTAGGG-3′ repeat to those previously studied. Next, synthesis of 5-repeat ODNs with an 8-oxoG or a Gh at the 5′-G of the first, third or fifth 5′-TTAGGG-3′ repeat was undertaken. The CD spectra for 5-repeat G4s with an 8-oxoG or a Gh was in contrast to the disruptive effect exhibited by the guanine lesions in the four-repeat quadruplex DNA. The CD data suggest that all 5-repeat G4 DNAs fold into hybrid-like structures, with the damage-containing repeat extruded from the fold when oxidized (Figure 6A–C). Furthermore, the T_m_ values for all 5-repeat G4 DNAs were similar, which support the idea that the damage is not impacting the global G4 structure (Figure 6D). Based on these results, we propose that damage (8-oxoG or Gh) in the 5′-repeat is part of a 5′-tail on a hybrid fold, while damage in the 3′-repeat is part of a 3′-tail on a hybrid fold (Figure 6A and C). Lastly, when the damage was placed in the middle repeat, the lesion-containing repeat is part of an extended edgewise loop that maintains the hybrid topology (Figure 6B).

The activities of NEIL1, NEIL2 and NEIL3 on the lesion-containing 5-repeat quadruplex DNA were then determined. Both NEIL1 and NEIL3 removed Gh (at the 5′ most site of a GGG triad) from all three 5-repeat quadruplexes (white bars) much faster than observed for the four-repeat quadruplex DNA (black bar) (Figure 6E and G). For example, under our assay conditions, NEIL1 removed more than 50% of the Gh from the three 5-repeat quadruplexes compared to 12.5% from the four-repeat quadruplexes. Surprisingly, while it was shown earlier that NEIL2 was not able to remove any hydantoins from the four-repeat quadruplexes, NEIL2 did remove Gh from the 5-repeat quadruplexes (compare Figures 3C and 6F). Thus it appears that the resistant lesions in quadruplex DNA may be removed through an alternative folding mechanism, which makes the lesion more accessible. This may be also true in cells considering the extensive availability of extra 5′-TTAGGG-3′ repeats in telomeres.

We also tested the activity of OGG1 on 8-oxoG-containing 5-repeat quadruplex DNA. OGG1 does not have activity on 5-repeat quadruplex DNA with 8-oxoG located at any of the three repeats (5′, middle and 3′) (Supplementary Figure S5, left). In comparison, OGG1 was able to remove 8-oxoG from the comparable duplex sequences efficiently and generated the correct products (Supplementary Figure S5, right). These data were expected, because our CD data showed that the 8-oxoG-containing repeat is extruded from quadruplex core to a single-stranded-like DNA region (Figure 6A–C), and OGG1 does not remove 8-oxoG from single-stranded DNA (19).

### APE1 cleaves furan at selected positions in Na^+^-coordinated quadruplex DNA but not in K^+^-coordinated quadruplex DNA

**Figure 7.**
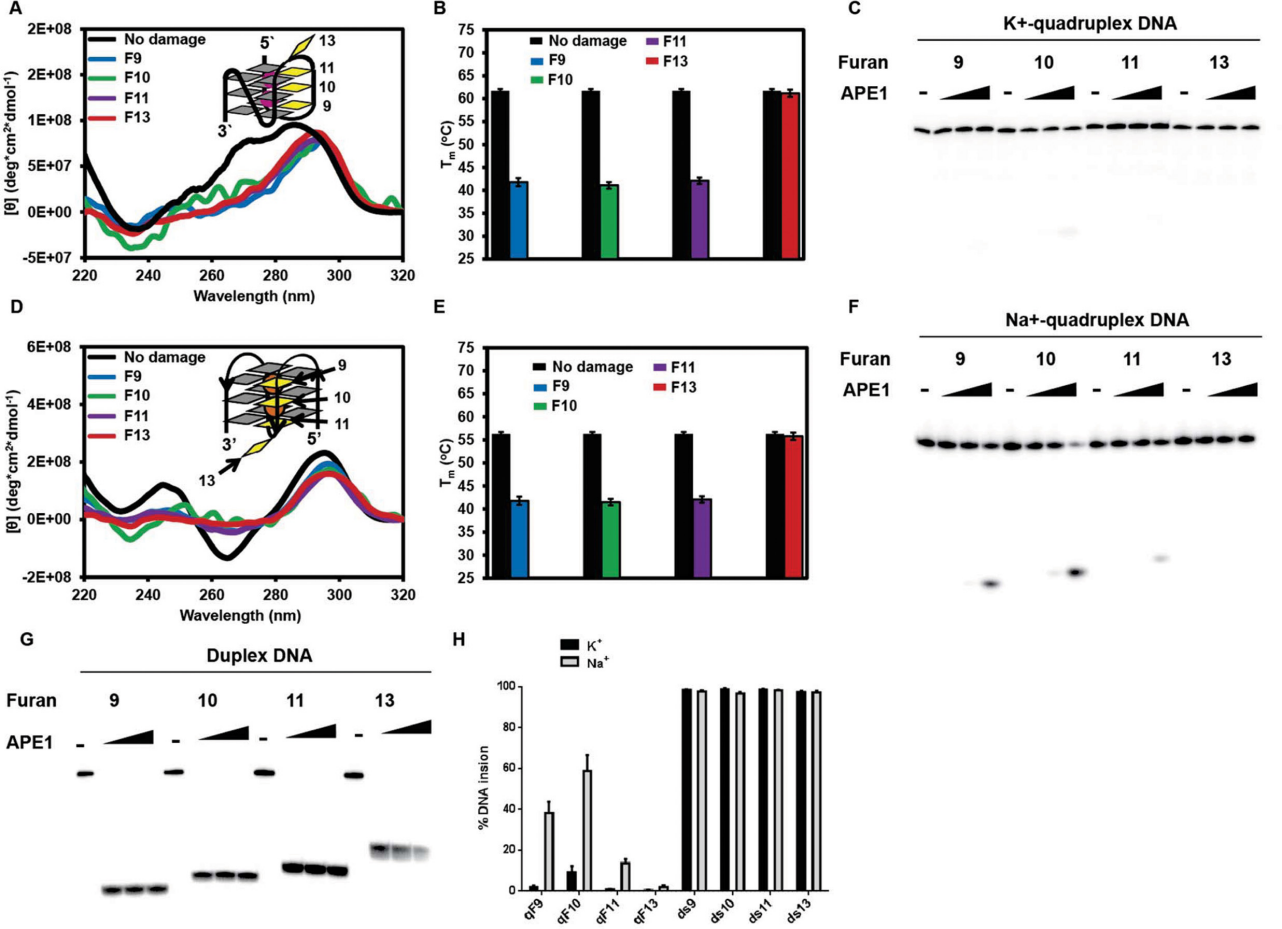
APE1 cleaves furan at selected positions in Na^+^-coordinated, but not K^+^-coordinated, telomeric quadruplex DNA. (**A, B**) CD spectra and T_m_ of furan-containing quadruplex DNA in KCl solution. (**C**) A representative gel shows that APE1 does not cleave furan from K^+^-coordinated quadruplex DNA. (**D, E**) CD spectra and T_m_ of furan-containing quadruplex DNA in NaCl solution. (**F**) A representative gel shows that APE1 cleaves furan from position 9 and 10 in Na^+^-coordinated quadruplex DNA. (**G**) APE1 activity on furan-containing control duplex substrates. Ten nanomolar of furan substrate and 1, 10 or 100 nM of APE1 were incubated at 37°C for 30 min. (**H**) Quantification of strand incision activity of APE1 on furan in K^+^-coordinated (black bars) and Na^+^-coordinated (grey bars) quadruplex DNA or control duplex DNA.

Abasic sites are one of the most abundant DNA lesions in genomes and their repair is essential for cell survival (82,83). This led us to study the activity of APE1 on abasic sites in quadruplex DNA. To measure the activity of APE1 on quadruplex DNA, ODNs with furan (a stable abasic site analog) were used. We first examined the structures of furan-containing telomeric sequences. Furan was introduced 5′, middle and 3′ of the G_­9_G_10_G_11_ stretch (F9, F10 and F11). We also looked at furan in a loop region (F13). In K^+^-containing buffer, all the furan-containing telomeric sequences presented the 295 nm maximum, indicating antiparallel quadruplex structures. However, F10 appeared to be a hybrid form of quadruplex (Figure 7A). Interestingly, there was no difference among the T_m_ values of F9, F10 or F11, which all exhibited a 20°C drop in T_m_ compared to the non-damage-containing hybrid quadruplex DNA (Figure 7B). This reduction of thermostability agrees with a previous report on abasic site-containing quadruplexes in K^+^ solution, except that a greater drop in T_m_ was observed when a center G was replaced with an abasic site (79).

We tested APE1 activity on these furan-containing quadruplexes formed in KCl buffer and found no cleavage of furan in any position. We tried enzyme concentrations up to 10-fold excess to substrate and there was still little detectable product (Figure 7C and H for quantification). In contrast, APE1 activity was robust on the corresponding duplex controls in K^+^ buffer, which went to completion under the same reaction conditions (Figure 7G and H).

Since secondary structure is required for the activity of APE1 on so-called ‘single-stranded’ DNA (84), we tested its activity on quadruplexes in Na^+^ solution. CD experiments were first carried out. All quadruplexes containing furan presented a dominant 295 nm antiparallel maximum, and in the case of F9 and F11, the G4s adopted a basket antiparallel structure in NaCl buffer, with the 295 nm maximum and 265 minimum (Figure 7D). F10 also appeared to be a basket quadruplex in Na^+^, but the minimum at 265 nm was not as prominent. F9, F10 and F11 all gave a T_m_ drop of ∼15°C in buffered NaCl solution, but there was also no difference in T_m_ among the three. In addition, the loop F13 had the same T_m_ as telomeric DNA without a lesion (Figure 7E).

We then tested the incision activity of APE1 on furan in Na^+^-coordinated quadruplexes. APE1 cleaved furan at positions 9 and 10 in Na^+^-coordinated quadruplexes (Figure 7F and H for quantification), although the activity was much slower than the optimal duplex DNA structure (Figure 7G and H for quantification). The quadruplex containing F10 was the best substrate of the four in Na^+^ buffer. By comparing the CD spectra of Na^+^- and K^+^-coordinated quadruplexes of F10, APE1 appeared to prefer the basket form of antiparallel quadruplex. Our results here are another example of secondary structure-dependent activity of APE1. We thus conclude that APE1 cleaves furan in Na^+^-coordinated telomeric basket quadruplex DNA but not in K^+^-coordinated hybrid quadruplex DNA.

### DNA glycosylases cannot remove 8-oxoG and Gh from K^+^-coordinated promoter quadruplexes/triplexes

Given the potential importance of quadruplexes in regulating gene transcription, we asked if glycosylases could remove guanine damages from the quadruplex DNA that potentially forms at promoter regions of critical genes. We chose the purine-rich nuclease hypersensitive elements of *c-MYC* and *VEGF* promoters as model sequences due to their critical cellular functions. Moreover, structures of quadruplexes formed by these two model sequences have been solved by NMR in KCl (85,86).

**Figure 8.**
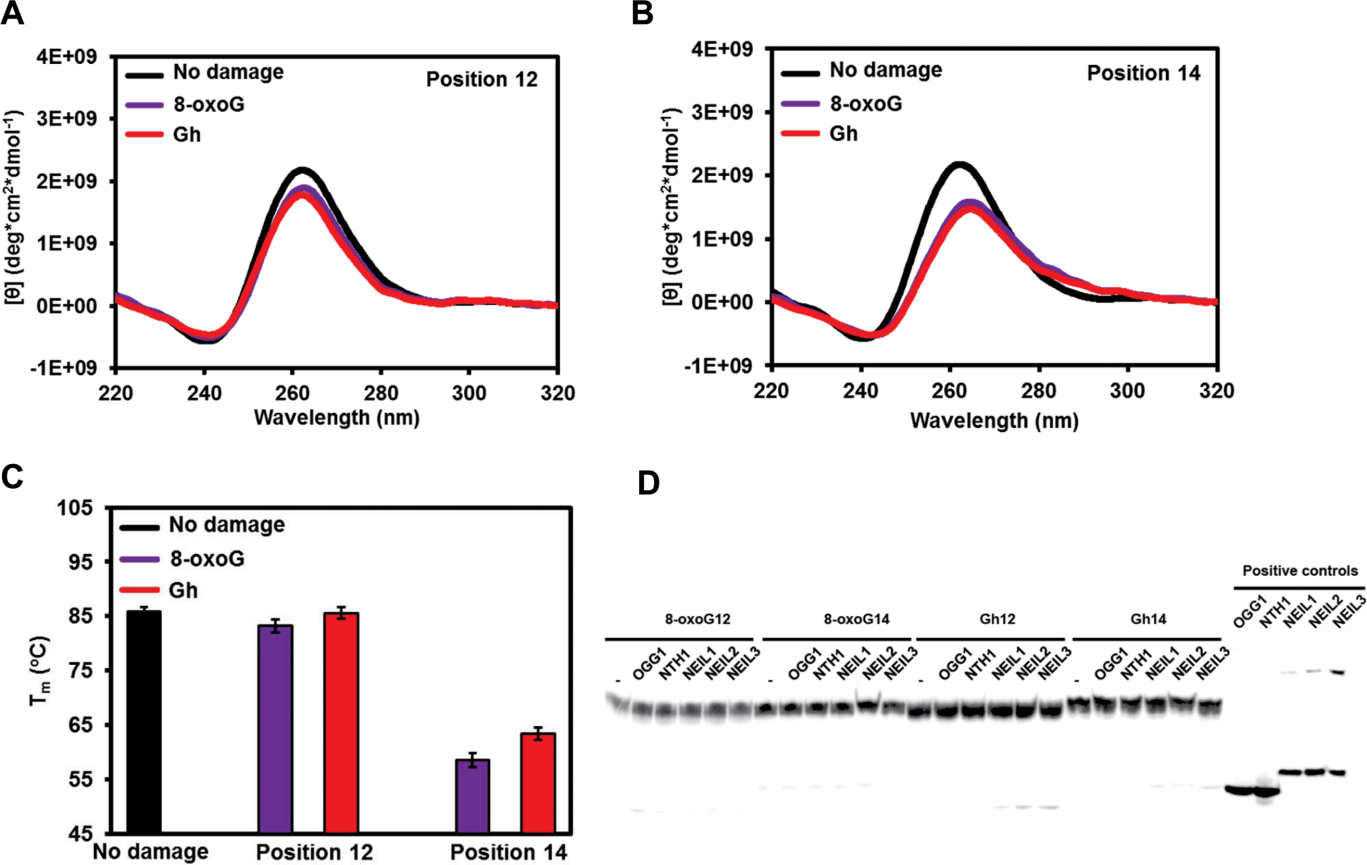
DNA glycosylases cannot remove 8-oxoG or Gh from K^+^-coordinated *VEGF* promoter quadruplexes/triplexes. (**A, B**) CD spectra of the *VEGF* promoter quadruplex in K^+^ solution with and without 8-oxoG or Gh lesions at position 12 (A) or 14 (B). (**C**) The T_m_ of *VEGF* promoter quadruplex in K^+^ solution with and without 8-oxoG or Gh lesions at position 12 or 14. (**D**) Glycosylases show no activity on K^+^-quadruplexes with the *VEGF* promoter sequence. Ten nanomolar substrate and 200 nM of enzyme were incubated at 37°C for 30 min.

We initiated our structural studies and enzymatic activity assays in a buffer containing KCl. The *VEGF* promoter sequence we used (VEGF-Pu22) forms a propeller-type parallel quadruplex structure in buffered K^+^ solution (86). An 8-oxoG or a Gh was introduced at positions 12 and 14, which are located in a loop and in a G-quartet core, respectively, in the above-mentioned structure. When the loop G (G12) was replaced by 8-oxoG or Gh in the *VEGF* promoter quadruplex, the *VEGF* sequences showed a positive 265 nm peak and a negative 240 nm peak (Figure 8A), suggesting an all-parallel, propeller-type quadruplex DNA structure. This is supported by the fact that there was no change in the T_m_ (Figure 8C). However, when a guanine in G-quartet (G14) was replaced by 8-oxoG or Gh, the CD spectrum redshifted and it had a nearly identical spectrum to the triplex DNA we previously reported (Figure 8B and (77)), suggesting formation of triplex DNA in the K^+^ buffer. There was also a T_m_ drop of 20°C when G14 was substituted by 8-oxoG14 or Gh14 in the *VEGF* quadruplex DNA (Figure 8C), which supports the triplex conformation of these two substrates.

In the case of the *c-MYC* promoter quadruplex, we used the Myc-2345 sequence, which contains the second, third, fourth and fifth G-tracks of the five-track 27-nt sequence (Pu27) of the *c-MYC* promoter (85). Phan *et al*. show that the Myc-2345 sequence also forms a propeller-type parallel G-quadruplex (85). Interestingly, replacing G with either 8-oxoG or Gh at either position 8 (in an exterior G-quartet) or position 11 (in a loop) did not cause any observable difference in the CD spectrum. All Myc-2345 sequences showed a positive 265 nm peak and a negative 240 nm peak (Supplementary Figure S6A and B), suggesting an all-parallel, propeller-type quadruplex DNA that is the same as the non-damage-containing sequence (85). The T_m_ studies of the *c-MYC* promoter quadruplexes agreed with their CD spectra. The T_m_ values of the lesion-containing Myc-2345 quadruplexes had the same values as the non-damage-containing quadruplexes (Supplementary Figure S6C). These data suggest that G8 will be pushed into a loop when damaged and G11 will be used to form a new quadruplex quartet, which is thermodynamically favored.

**Figure 9.**
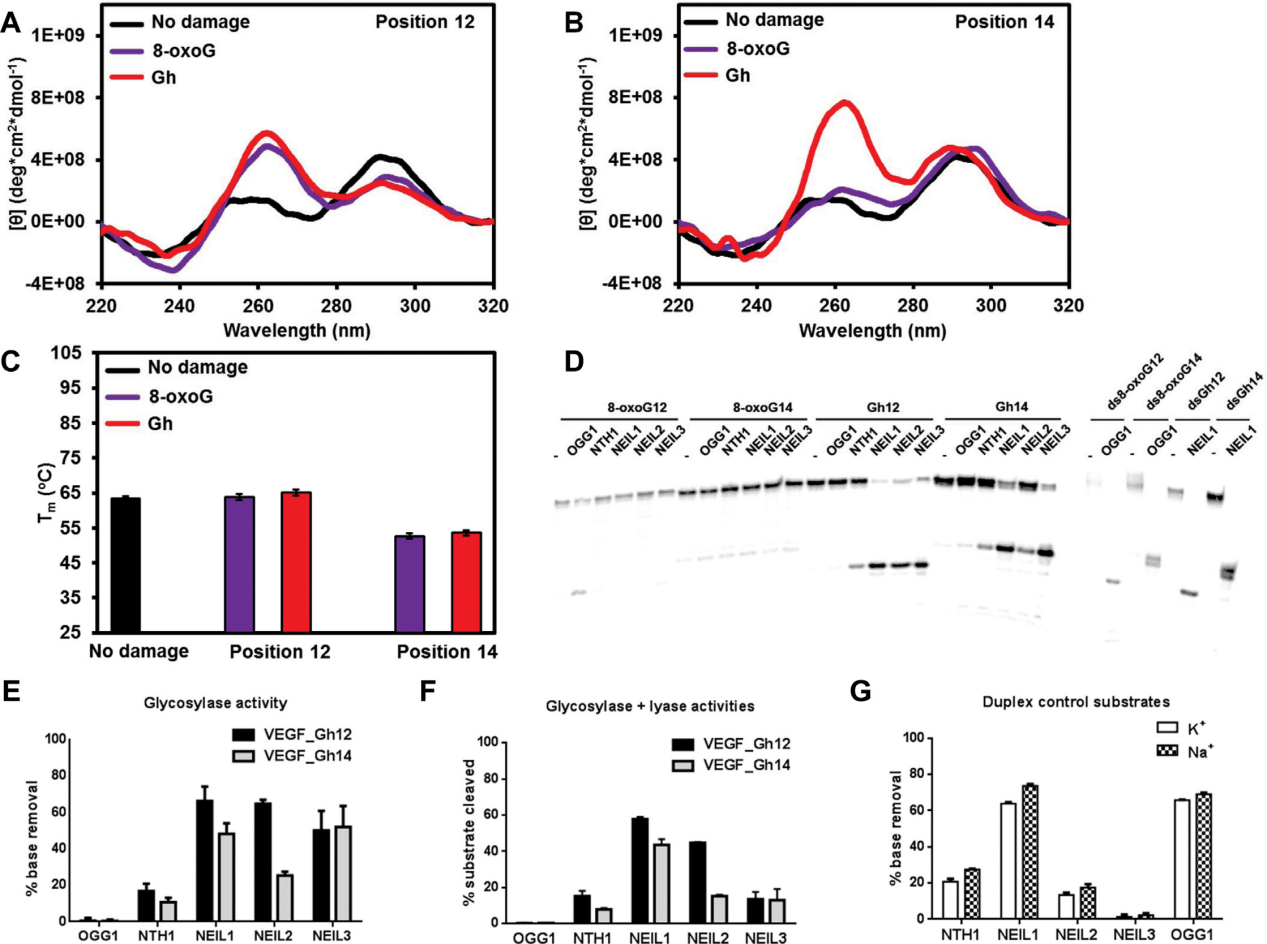
The NEIL glycosylases remove Gh from Na^+^-coordinated promoter quadruplexes. (**A, B**) CD spectra of the *VEGF* promoter quadruplex in Na^+^ solution with and without a lesion at position 12 (A) or 14 (B). (**C**) T_m_ of the *VEGF* promoter quadruplex in Na^+^ solution with and without 8-oxoG or Gh lesions at position 12 or 14. (**D**) A representative gel of a glycosylase assay on Gh in a Na^+^-coordinated *VEGF* promoter quadruplex showing that the NEIL glycosylases remove Gh from quadruplex structures. (**E**) Quantification of glycosylase activity on Gh in the *VEGF* promoter quadruplex. (**F**) Quantification of glycosylase plus lyase activity on Gh-containing *VEGF* promoter quadruplexes. (**G**). Comparison of the glycosylase activity of each glycosylase on duplex DNA in K^+^ and Na^+^ reaction buffers where no significant difference was found. For reaction with quadruplex DNA, 10 nM substrate and 200 nM of enzyme were used. For duplex DNA controls, 10 nM substrate and 10 nM enzyme were used. Reactions were incubated at 37°C for 30 min and quenched with NaOH and heating to measure glycosylase activity, or quenched with formamide/EDTA to measure glycosylase plus lyase activity.

Glycosylase activity assays were carried out on these substrates. In K^+^ buffer, 8-oxoG in the *VEGF* promoter quadruplex DNA could not be removed by any of the human glycosylases, including OGG1 (Figure 8D), similar to the results obtained with the telomeric quadruplexes. In addition, the Gh in the *VEGF* promoter quadruplex could not be removed by any glycosylase (Figure 8D). The positive controls showed that the enzymes we used were active (Figure 8D, right), and mass spectrometry data (Supplementary Table S1) and cleavage of duplex ODNs experiments (see Figure 9D and below) confirmed that the ODNs we used here contained the correct damages at the correct positions. In duplex DNA with the same sequences, 8-oxoG and Gh could be appropriately removed by OGG1 and NEIL1, respectively (Figure 9D, right). Similar results were observed for the *c-MYC* promoter quadruplex. No glycosylase was able to remove 8-oxoG or Gh from the *c-MYC* promoter quadruplex DNA in K^+^ solution (Supplementary Figure S6D). We thus conclude that glycosylases cannot remove guanine oxidation damages from K^+^-coordinated, parallel quadruplex/triplex DNA containing the *c-MYC* or the *VEGF* promoter sequences.

### The NEIL glycosylases do have glycosylase and lyase activities on Gh in Na^+^-coordinated promoter quadruplexes

Because the promoter sequences can adopt different quadruplex structures in K^+^ and Na^+^ buffers (87), we extended our promoter quadruplex study to include buffered Na^+^ solutions. CD spectra and T_m_ studies of the *VEGF* promoter sequences were carried out. First, the T_m_ of Na^+^ quadruplexes were lower than the K^+^ quadruplexes of the same sequence (Figure 9C), indicating Na^+^ quadruplexes were less stable. Second, the CD spectra were also very different between the Na^+^ and K^+^ quadruplexes (Figure 9A and B). The Na^+^ quadruplexes displayed an extra positive peak at 295 nm that was absent from the K^+^ quadruplexes. The combination of 295 positive, 265 positive and 240 negative peaks suggested that there was a mixture of parallel and antiparallel strand orientations in the Na^+^ buffer. A similar result was observed when a guanine was replaced by inosine in a telomeric quadruplex (87).

To our surprise, we observed very different results when glycosylase activity assays were done in Na^+^-coordinated quadruplex DNA. All three NEIL glycosylases were able to remove Gh from *VEGF* promoter quadruplex DNA efficiently at both positions (Figure 9D and E, Gh12 and Gh14). NTH1 had only marginal activity. We also found that OGG1 had slow activity on 8-oxoG in the *VEGF* promoter quadruplex at position 12 (Figure 9D).

In order to answer the question as to whether the different activities of glycosylases on K^+^- and Na^+^-coordinated quadruplexes was an effect of cations, glycosylase activities of the five enzymes on *VEGF* duplex DNA (containing 8-oxoG12 or Gh12) were compared in buffered K^+^ or Na^+^ solutions (Figure 9G). NEIL1, NEIL2 and NTH1 showed no significant difference in activity on duplex-Gh12 in K^+^ or Na^+^ solutions, and the cation used did not affect the activity of OGG1 on the duplex-8-oxoG12 substrate. NEIL3 showed very little activity on duplex DNA in both K^+^ and Na^+^ solutions as expected. These observations suggest that the *VEGF* promoter sequence adopts different quadruplex structures in K^+^ and Na^+^ that are recognized differently by the glycosylases. As shown above, the *VEGF* promoter sequence in Na^+^ showed a mixture of antiparallel and parallel strand orientations, while in K^+^ it presented an all-parallel quadruplex structure (8-oxoG12 and Gh12) or a triplex structure (8-oxoG14 and Gh14). We reason that NEIL glycosylases primarily recognize damages in a *VEGF* promoter quadruplex that has an antiparallel strand arrangement.

We also asked if the NEIL glycosylases have lyase activity on promoter quadruplex DNA after base removal. The reactions were started with Gh-containing quadruplexes as substrates and were stopped by formamide/EDTA stoppage buffer without NaOH. NEIL1 and NEIL2 had efficient lyase activity on quadruplex DNA producing strand breaks after base removal (Figure 9F and Supplementary Figure S8A). On the other hand, there was very little strand break product (13%) when NEIL3 was incubated with the Gh-containing *VEGF* promoter quadruplex (Figure 9F), where 52% of Gh had been removed generating AP sites (Figure 9E), indicating that NEIL3 has slow lyase activity on AP sites in the *VEGF* promoter quadruplex. When we added APE1 to the NEIL3 reaction, we did not see a significant increase in the strand incision product, indicating that APE1 also does not cleave AP sites in *VEGF* promoter quadruplexes (Supplementary Figure S8A).

We have done the same sets of experiments using the *c-MYC* promoter quadruplex in Na^+^ buffer and found that the CD spectra of the *c-MYC* promoter sequence were very different from *VEGF* promoter sequence. All *c-MYC* sequences with or without a lesion gave a 265 positive peak and a 240 negative peak, suggesting an all-parallel, propeller-type of quadruplex (Supplementary Figure S7A and B). In addition, there was also no significant difference in melting temperatures among these quadruplexes (Supplementary Figure S7C). Moreover, the activity assays showed that as with the telomeric and *VEGF* quadruplex structures, the glycosylases were unable to remove 8-oxoG from Na^+^-coordinated *c-MYC* promoter quadruplex (Supplementary Figure S7D). Although we did observe some activity of the NEIL glycosylases on Gh-containing *c-MYC* quadruplex substrates, the rate was much slower than on those formed by *VEGF* sequences (both glycosylase activity alone (Supplementary Figure S7E) and glycosylase plus lyase activity (Supplementary Figure S7F)). These data support the conclusion that the NEIL glycosylases primarily remove hydantoin lesions from promoter quadruplex DNA having an antiparallel strand arrangement.

## DISCUSSION

We have studied the structures and thermostability of telomeric quadruplexes containing guanine oxidative damages (8-oxoG, (*S*)-Sp, (*R*)-Sp and Gh) at multiple positions. The guanine lesions reduce the thermostability of telomeric quadruplex DNA and alter its folding in a lesion position-dependent manner, while the lesion type does not play a significant role. The general rule is that oxidation in the middle quartet results in the most disruptive effect to the quadruplex structure, which is consistent with several other studies when guanine was substituted with an abasic site or 8-oxoguanine (52,79). Here we propose the most reasonable structure for each lesion-containing substrate, based on their CD spectra and supported by T_m_ studies. The CD spectra of lesion-containing telomeric quadruplexes were not all perfectly characteristic of a particular quadruplex structure due to their dynamic nature in solution. Moreover, the fact that 8-oxoG placed in an exterior quartet of the human telomere sequence led to a structure that was so poorly folded that suitable NMR signals could not be obtained (88) is consistent with our results.

Because guanine has the lowest redox potential of the native bases, 8-oxoG is one of the most prevalent oxidative lesions in cells (89). 8-oxoG can alter telomere sequences by mispairing with A (90) and it also inhibits shelterin proteins from binding to telomere DNA (53). Further oxidation of 8-oxoG results in the formation of hydantoins that may block replication of telomere DNA (11). We show here and previously (25) that 8-oxoG cannot be removed from telomeric quadruplex DNA by human DNA glycosylases, including OGG1, which provides one explanation of why telomeres contain more 8-oxoG lesions than the rest of the chromosome (91). Upon further oxidation 8-oxoG to hydantoins, oxidized guanines can then be removed by the NEIL1 and NEIL3 glycosylases, as shown in this study.

We also show that abasic sites in quadruplex DNA can be hydrolyzed by the lyase activity of NEIL1 and NEIL3, although the rate of NEIL3 is slow (Supplementary Figure S3). NEIL1 and NEIL3 exhibited most of their activities at position 10 and position 11, which mirrored their glycosylase activity that was presumably rate-limiting; therefore, one could not make direct comparison of the lyase activities among positions or between enzymes. We could not determine if NEIL2 has lyase activity on abasic sites in G4 DNA, since we started with Gh-containing quadruplex DNA, which is not a substrate for NEIL2 (Figure 3C).

We previously showed that the NEIL3 and NEIL1 glycosylases were the principal enzymes that removed hydantoin lesions from one position in telomeric quadruplex DNA (25). We also showed (50) that oxidation of quadruplex DNA results in hydantoins as major products at the outer quartets (5′ followed by 3′ of GGG); however, our current study shows that the 5′ hydantoins are removed much less efficiently by the NEIL glycosylases. The alternative folding model of the 5-repeat quadruplex provides a solution to this dilemma. When extra 5′-TTAGGG-3′ repeats are available, which is always the case considering the long human telomere repeats, damage-containing 5′-TTAGGG-3′ stretches may be looped out from the quadruplex core and processed by the NEIL glycosylases. Repair of 8-oxoG in quadruplexes may also utilize this alternative folding mechanism, when the looped out region is annealed to a complementary strand.

A number of recent studies have shown that BER enzymes are required for telomere homeostasis. Mouse embryonic fibroblast cells that lack either NTH1 or OGG1 glycosylase show telomere defects including telomere shortening and loss, concurrent with increased numbers of DNA base damages in telomere DNA (92,93). We did not find significant activity of NTH1 or OGG1 on telomeric quadruplex DNA in the current study or in our previous study (25), suggesting these enzymes function primarily on telomere duplex DNA. The same interpretation applies to APE1, except that we showed APE1 could process furan at selected positions in an Na^+^-coordinated quadruplex DNA albeit at a slow rate. Madlener *et al*. have recently shown that APE1 is required for telomere maintenance (94). Taken together, our data suggest that NEIL1 and NEIL3 are the glycosylases that remove damages from telomeric quadruplex DNA, leading us to speculate on their function in telomeres. Our unpublished data show that knock down of NEIL3 in human cells results in telomere defects, suggesting that repair of quadruplex damage may be required for telomere maintenance, given the fact that the activity of NEIL3 on duplex telomeric DNA is slow. It will be interesting to see if cells where NEIL1 is knocked down also develop telomere defects. Nevertheless, it is clear that the BER glycosylases are involved in the repair of telomere DNA (both duplex and quadruplex) to ensure their proper function.

Several bioinformatics studies reported the enrichment of potential quadruplex-forming sequences at promoters of genes (35,36), which led to a model of transcriptional control via assembly and disassembly of quadruplex DNA (34). This model is supported by transcription studies of a number of biologically important promoters, including *c-MYC* and *VEGF* (59,67). BER enzymes, OGG1 glycosylase in particular, have been implicated in gene transcription regulation by altering local DNA secondary structure and chromosomal arrangement (95). Gillespie's group showed that G4-forming sequences are targets of oxidation in hypoxia-induced signaling, after which the BER enzymes, OGG1 and APE1, are recruited to these damaged G4 sequences (96). These same researchers also found that oxidative modifications of the hypoxia response elements coincided with the onset of mRNA accumulation of hypoxia-inducible genes, including the *VEGF* gene (97). Based on these observations, they hypothesized a model of controlled DNA damage and repair to regulate gene expression, by altering transcription factor binding and increasing DNA/chromatin flexibility (97,98). However, no direct link has been made between damage repair and secondary structure changes.

Here, we provide biochemical evidence that links damage base removal followed by strand breakage to potential gene regulation. The folding of promoter quadruplex DNA is dependent on coordinating ions, which leads to differential accessibility of glycosylases to the lesions. The NEIL glycosylases were able to remove hydantoin lesions from Na^+^-coordinated (antiparallel), but not from K^+^-coordinated (parallel) quadruplex DNA, which leads us to hypothesize a switch model of transcription via the differential actions of glycosylases on lesions in quadruplex DNA structures (Supplementary Figure S9).

NEIL1, NEIL2 and NEIL3 remove damaged bases from Na^+^-coordinated antiparallel quadruplex DNA, and NEIL1 and NEIL2 are able to cleave the abasic sites produced. The strand break created by NEIL2 leads to the collapse of quadruplex DNA (Supplementary Figure S8B). Interestingly, NEIL2 is linked to transcription-coupled repair (30). Our data suggest that NEIL2 may also function as a transcription initiator for certain genes by removing quadruplex DNA structures at their promoters. We could not determine if the quadruplex structure collapses after NEIL1 lyase activity, because the substrate remained bound to the enzyme (Supplementary Figure S8B).

Considering that K^+^ is the major cation in cells, the parallel quadruplex structure is presumably the dominant structure formed *in vivo*, which sets the promoter to an ‘OFF’ position. Conversely, using structure-specific antibodies, Schaffitzel *et al*. found that the antiparallel quadruplex was the dominant form found in *Stylonychia lemnae* macronuclei (99). In addition, quadruplex folding proteins may be involved in the parallel/antiparallel quadruplex switch model. For example, the telomere end-binding proteins promote formation of antiparallel quadruplex DNA in *Stylonychia* (100). On the other hand, the C-terminus of nucleolin promotes the formation of a parallel G-quadruplex at the *c-MYC* promoter and inhibits its promoter activity (101). We argue that the local concentration of K^+^ and Na^+^ may vary around G4 sequences of interest, which may dictate which quadruplex structure is formed and whether the lesion in the quadruplex is accessible to glycosylases, which will ultimately determine the status of the promoter.

In conclusion, we show here that the NEIL glycosylases remove oxidized guanine lesions from quadruplex DNA structures formed by telomere sequences as well as *VEGF* and *c-MYC* promoter sequences. These data suggest that the NEIL glycosylases may function in telomere maintenance by initiating BER in quadruplex DNA. Our data also provide a biochemical explanation as to how DNA glycosylases may function in gene regulation by acting on quadruplex DNA formed at promoter regions, although *in vivo* experimental data are necessary to support this model.

## SUPPLEMENTARY DATA

Supplementary Data are available at NAR Online.

SUPPLEMENTARY DATA
