# The NEIL glycosylases remove oxidized guanine lesions from telomeric and promoter quadruplex DNA structures

**DOI:** 10.1093/nar/gkv673

**Published:** 2015-06-27

**Authors:** Jia Zhou, Aaron M. Fleming, April M. Averill, Cynthia J. Burrows, Susan S. Wallace

*Nucl. Acids Res*. 43 (8): 4039–4054. doi: 10.1093/nar/gkv252

The authors wish to draw attention to an error in their published article.

In Figure 1C, the red structure in the third G-quartet should be guanidinohydantoin (Gh) instead of spiroiminodihydantoin (Sp). A new Figure 1 with Gh in G-quartet is included below.

**Figure 1.**
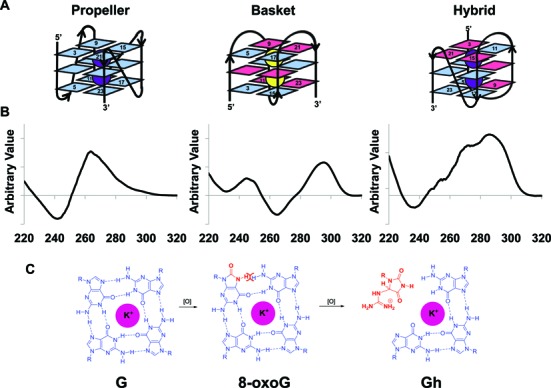
Folding, CD spectra and guanine oxidation of quadruplex DNA. (**A**) Folding of parallel propeller, antiparallel basket and hybrid (type 2) quadruplex DNA. (**B**) Representative CD spectrum of each quadruplex DNA. In a parallel quadruplex, all four strands point in one direction and the neighboring strands are connected with double reversal loops. The CD spectrum of a parallel quadruplex features a 265 nm maximum and a 240 nm minimum. The basket antiparallel quadruplex DNA has neighboring strands running in opposite directions and connected with two lateral loops and a diagonal loop. This structure features a 295 nm maximum and a 265 nm minimum. A hybrid (type 2) quadruplex has mixed strand directionalities and presents a 295 nm maximum, a 270 nm shoulder and a 235 nm minimum (see (46)). (**C**) Chemical structures of guanine (G), 8-oxoguanine (8-oxoG) and guanidinohydantoin (Gh) in the context of a G quartet.

The findings and conclusion of the article remain valid.

The authors apologise to readers for this error and any inconvenience caused.

## Supplementary Material

SUPPLEMENTARY DATA

