# Supplementary material for: The NEIL glycosylases remove oxidized guanine lesions from telomeric and promoter quadruplex DNA structures
Source: Nucleic Acids Res. 2015 Mar 26;43(8):4039–54. doi: 10.1093/nar/gkv252 (PMC4417164; doi:10.1093/nar/gkv252)
Supplement: SUPPLEMENTARY DATA [file supp_43_8_4039__index.html]

The NEIL glycosylases remove oxidized guanine lesions from telomeric and promoter quadruplex DNA structures — The NEIL glycosylases remove oxidized guanine lesions from telomeric and promoter quadruplex DNA structures — SUPPLEMENTARY DATA 

# The NEIL glycosylases remove oxidized guanine lesions from telomeric and promoter quadruplex DNA structures

## SUPPLEMENTARY DATA

**Files in this Data Supplement:**

- SUPPLEMENTARY DATA
