# Supplementary material for: The NEIL glycosylases remove oxidized guanine lesions from telomeric and promoter quadruplex DNA structures
Source: Nucleic Acids Res. 2015 Mar 26;43(8):4039–54. doi: 10.1093/nar/gkv252 (PMC4417164; doi:10.1093/nar/gkv252)
Supplement: SUPPLEMENTARY DATA [file supp_gkv252_nar-00331-f-2015-File011.pdf]

The NEIL glycosylases remove oxidized guanine lesions from telomeric and promoter quadruplex DNA structures

Jia Zhou[1], Aaron M. Fleming[2], April M. Averill[1], Cynthia J. Burrows[2*], and Susan S. Wallace[1*]

[1] Department of Microbiology and Molecular Genetics, University of Vermont, Burlington, Vermont 05405, United States

[2] Department of Chemistry, University of Utah, Salt Lake City, Utah 84112, United States

The authors wish it to be known that, in their opinion, the first two authors should be regarded as joint First Authors.

Supplemental Information

Supplemental Methods

Supplemental Table and Figure Legends

Supplemental References

Supplemental Table S1

Supplemental Figures S1-S9

Synthesis and Purification of oligodeoxyribonucleotides (ODN)

All 8-oxoG-containing ODNs were synthesized and deprotected by the DNA-peptide core facility at the University of Utah following the manufacturer's protocols (Glen Research, Sterling, Virginia). The crude samples were purified by semi-preparative ion exchange HPLC running the following mobile phases: A = 1.5 M LiOAc (pH 7.0) in 10% MeCN and 90% ddH$_2$O, and B = 10% MeCN and 90% ddH$_2$O while running a flow rate of 3 mL/min and monitoring the absorbance at 260 nm. Purification salt was removed by dialysis against ddH$_2$O for 36 hr using a 3500 molecular weight cutoff (MWCO) membrane cassette. The purified samples were used in the synthesis of Sp and Gh via the following methods. Synthesis of Gh was achieved by mixing a 20 μM solution of 8-oxoG-containing ODN in ddH$_2$O at 20°C with 120 μM K$_2$IrBr$_6$, while letting the reaction sit for 30 min. Synthesis of Sp was achieved by mixing a 20 μM solution of 8-oxoG-containing ODN in 10 mM NaP$_i$ (pH 8.0) buffer preincubated at 45°C, after which 120 μM K$_2$IrBr$_6$ was added, while letting the reaction sit for 30 min. The samples were purified using an analytical ion-exchange HPLC setup running the following mobile phases: A = 1 M LiCl, 25 mM Tris (pH 8.0) in 10% MeCN and 90% ddH$_2$O, and B = 10% MeCN and 90% ddH$_2$O while running a flow rate of 1 mL/min and monitoring the absorbance at 260 nm. Purification salts were removed by dialysis against ddH$_2$O for 36 hr using 3500 MWCO membrane cassettes. Product purity was determined by analytical ion-exchange HPLC and product identity was determined by ESI-MS (Figure S1 and Table S1). Oligodeoxyribonucleotides were quantified by NanoDrop spectrophotometry using their extinction coefficients.

Supplemental Table and Figure Legends

Table S1. HPLC and ESI-MS analysis of the synthesized oligodeoxyribonucleotides. The (*) indicates that this value was determined on a mixture of the Sp diastereomers.

Figure S1. HPLC analysis of the lesion-containing telomeric G4 and promoter G4 sequences. (A) HPLC traces of hydantoin-containing 4-repeat telomere sequences. The (*) indicates that the two peaks observed for Gh represent the interchangeable R and S diastereomers of Gh (1). (B) HPLC traces of 5-repeat telomere sequences with an 8-oxoG or a Gh. (C) HPLC traces of the *VEGF* promoter sequence with an 8-oxoG or a Gh at position 12 or position 14. (D) HPLC traces of the c-*MYC* promoter sequence with an 8-oxoG or a Gh at position 8 or position 11.

Figure S2. NEIL1 and NEIL3 remove *(S)*-Sp and *(R)*-Sp from quadruplex/triplex DNA. NEIL1, human NEIL3 and mouse NEIL3, but not OGG1, NEIL2 or NTH1, remove (*S*)-Sp (A) and (*R*)-Sp (B) from quadruplex/triplex DNA. 10 nM of each substrate was incubated with 200 nM of each glycosylase at room temperature for 30 min, and the reaction was stopped by adding NaOH and heating.

Figure S3. NEIL1 and NEIL3 exhibit lyase activity on an AP site in telomeric quadruplex DNA. 10 nM Gh-containing quadruplex DNA was incubated with 10 nM of enzyme at room temperature for 30 min. The reactions were stopped by formamide/EDTA buffer to visualize glycosylase plus lyase activities. Lyase activity of NEIL1 (A), NEIL2 (B), and NEIL3 (C) on abasic sites in telomeric quadruplex DNA after removal of Gh is shown (mean and standard deviation from three experiments).

Figure S4. Reaction time courses for NEIL3 k_{obs} measurements. NEIL3 shows poor cleavage of the 5' lesions (Gh and (*R*)-Sp) in quadruplex DNA (A and B), which is not the case for duplex DNA (C and D). All reactions contain 10 nM substrate and 100 nM active NEIL3 enzyme. Reactions were stopped by mixing with NaOH and heating after incubating at room temperature at the indicated time points.

Figure S5. OGG1 does not remove 8-oxoG from quadruplex DNA formed by 5-repeat telomere sequences. Left, 5-repeat telomere quadruplex DNA. Right, corresponding 5-repeat telomere duplex DNA controls. 10 nM of 5-repeat telomere quadruplex DNA was incubated with 100 nM of OGG1 at 37°C for 10 min. Reactions were stopped by adding NaOH and heating.

Figure S6. Glycosylases cannot remove 8-oxoG or Gh from K^+-coordinated *c-MYC* promoter quadruplexes. The CD spectra of *c-MYC* promoter quadruplexes in K^+ solution with and without a lesion at position 8 (A) and position 11 (B). (C) T_m values of the *c-MYC* promoter quadruplexes in K^+ solution. (D) Glycosylase assays with K^+-quadruplexes with the *c-MYC* promoter sequence. 10 nM of substrate and 200 nM of enzyme were incubated at 37°C for 30 min.

Figure S7. The NEIL glycosylases remove Gh from the Na^+-coordinated *c-MYC* promoter quadruplexes. The CD spectra of *c-MYC* promoter quadruplexes in Na^+ solution with and without a lesion at position 8 (A) and position 11 (B). (C) T_m values of the *c-MYC* promoter quadruplexes in Na^+ solution. (D) Glycosylase activity on Na^+-quadruplexes with the *c-MYC* promoter sequence. (E) Quantification of glycosylase activity on Gh-containing quadruplexes with the *c-MYC* promoter sequence. (F) Quantification of glycosylase plus lyase activity on Gh-containing quadruplexes with the *c-MYC* promoter sequence. 10 nM of substrate and 200 nM of enzyme were incubated at 37°C for 30 min.

Figure S8. NEIL1 and NEIL2 show glycosylase plus lyase activities on promoter quadruplex DNA and the lyase activity disrupts quadruplex folding. 10 nM Gh-containing promoter quadruplex DNA was incubated with 100 nM of enzyme at 37°C for 30 min. (A) The reactions were stopped by formamide/EDTA buffer to visualize glycosylase plus lyase activities on a urea denaturing gel. Quantifications of these images were shown in Figure 9F and Figure S7F. (B) The same reactions were mixed with glycerol (5% final) and load to a native gel to visualize quadruplex DNA disruption.

Figure S9. A model for promoter activation by the glycosylase action on promoter quadruplex DNA. When a quadruplex is present at a promoter (i.e., *VEGF* promoter), transcription of the downstream gene

is off. Oxidative stress induces oxidative damages in the susceptible G-rich quadruplex DNA structure. The oxidation of guanine, Na^+/K^+ exchange, or quadruplex folding proteins may induce the quadruplex to a topology that is recognizable by glycosylases. Glycosylases remove the damaged base from the quadruplex DNA and break the DNA backbone, which causes collapse of the quadruplex DNA structure and allows activation of the promoter.

Supplemental References

1. Ye, Y., Muller, J. G., Luo, W., Mayne, C. L., Shallop, A. J., Jones, R. A., and Burrows, C. J. (2003) Formation of 13C-, 15N-, and 18O-labeled guanidinohydantoin from guanosine oxidation with singlet oxygen. Implications for structure and mechanism. *J Am Chem Soc* **125**, 13926-13927

Table S1

Sample	Sequence	Calculated Mass, Da	Experimental Mass, Da
Tel	5'-TA GGG TTA GGG TTA GGG TTA GGG TT	7878.2	7878.4
Tel-9	5'-TA GGG TTA **X**GG TTA GGG TTA GGG TT		
**X =**			
Gh		7884.2	7884.6
(*S*)-Sp		7910.2*	7909.6
(*R*)-Sp			
8-oxoG		7894.2	7894.4
Tel-10	5'-TA GGG TTA G**X**G TTA GGG TTA GGG TT		
**X =**			
Gh		N.D.	N.D.
(*S*)-Sp		N.D.	N.D.
(*R*)-Sp		N.D.	N.D.
8-oxoG		N.D.	N.D.
Tel-11	5'-TA GGG TTA GG**X** TTA GGG TTA GGG TT		
**X =**			
Gh		N.D.	N.D.
(*S*)-Sp		N.D.	N.D.
(*R*)-Sp		N.D.	N.D.
8-oxoG		N.D.	N.D.
5-repeat	5'-TA GGG TTA GGG TTA GGG TTA GGG TTA GGG TT	9787.4	9787.2
5-repeat with lesions at 5' repeat			
**X=**	5'-TA **X**GG TTA GGG TTA GGG TTA GGG TTA GGG TT		
8-oxoG		9803.4	9803.2
Gh		9793.4	9793.6
5-repeat with lesions at middle repeat			
**X=**	5'-TA GGG TTA GGG TTA **X**GG TTA GGG TTA GGG TT		
8-oxoG		N.D.	N.D.
Gh		9793.4	9793.7
5-repeat with lesions at 3' repeat			
**X=**	5'-TA GGG TTA GGG TTA GGG TTA GGG TTA **X**GG TT		
8-oxoG		N.D.	N.D.
Gh		9793.4	9793.6

VEGF (Pu22)	5'-CG GGG C GGG CC GGGGG C GGG GT	6945.5	6945.8
Position 12			
**X**=	5'-CG GGG C GGG CC **X**GGGG C GGG GT		
8-oxoG		6970.5	6971.4
Gh		6960.5	6960
Position 14			
**X**=	5'-CG GGG C GGG CC GG**X**GG C GGG GT		
8-oxoG		6970.5	6971.2
Gh		6960.5	6960.8
c-MYC (Myc2345)	5'-TGA GGG TGGGG A GGG T GGGG AA	7040.6	7040.8
Position 8			
**X**=	5'-TGA GGG T**X**GGG A GGG T GGGG AA		
8-oxoG		7056.6	7056.8
Gh		7046.6	7047.1
Position 11			
**X**=	5'-TGA GGG TGGG**X** A GGG T GGGG AA		
8-oxoG		7056.6	7056.8
Gh		7046.6	7047.2

Figure S1

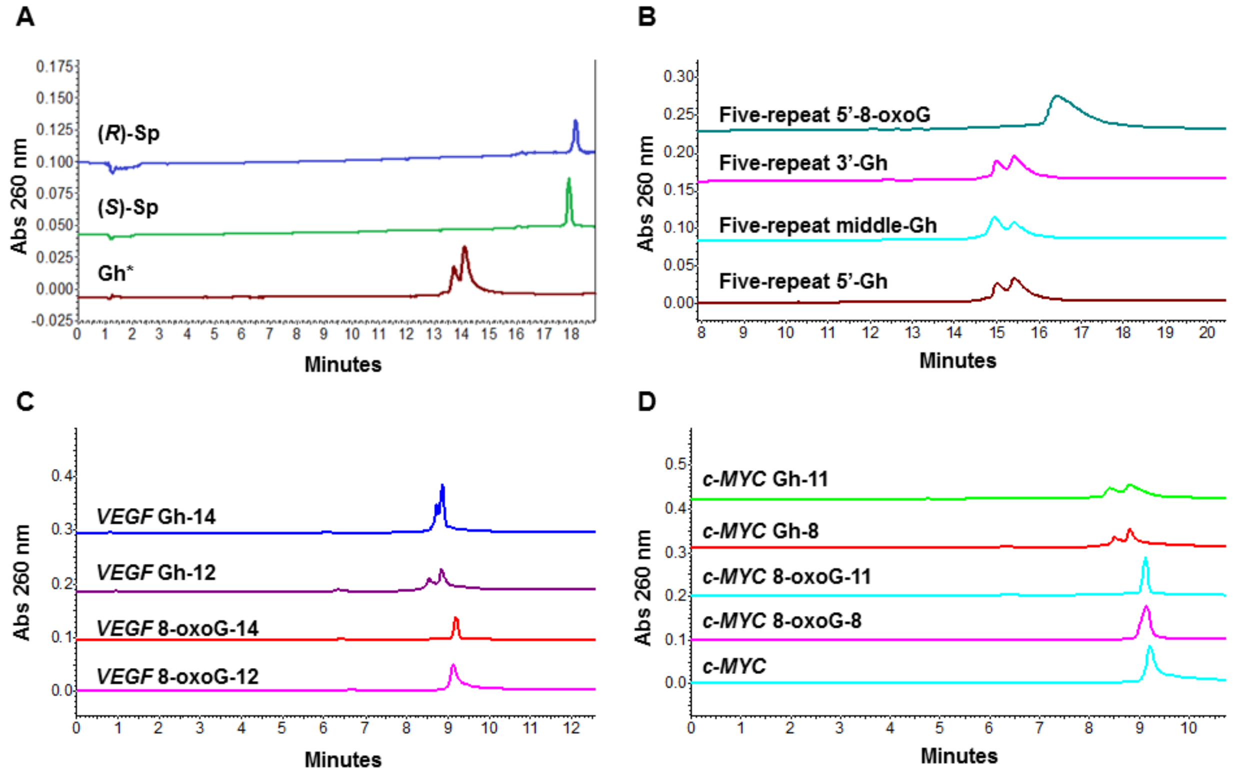
A
(R)-Sp
(S)-Sp
Gh*
Abs 260 nm
Minutes
B
Five-repeat 5'-8-oxoG
Five-repeat 3'-Gh
Five-repeat middle-Gh
Five-repeat 5'-Gh
Abs 260 nm
Minutes
C
VEGF Gh-14
VEGF Gh-12
VEGF 8-oxoG-14
VEGF 8-oxoG-12
Abs 260 nm
Minutes
D
c-MYC Gh-11
c-MYC Gh-8
c-MYC 8-oxoG-11
c-MYC 8-oxoG-8
c-MYC
Abs 260 nm
Minutes

Figure S2

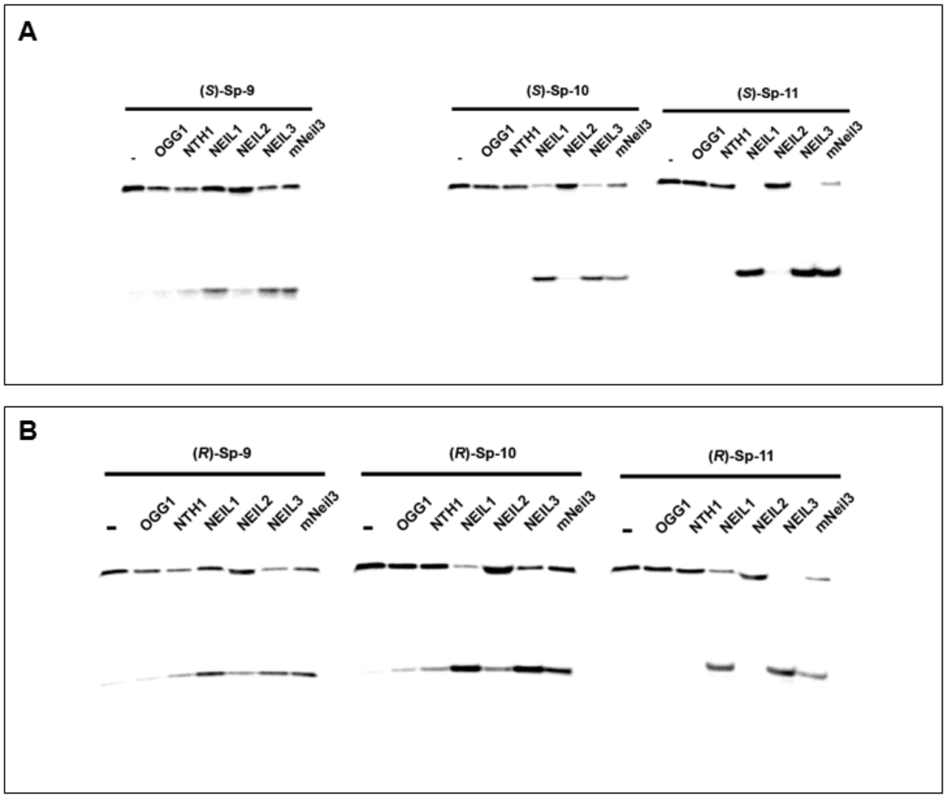
A
(S)-Sp-9
-
OGG1
NTH1
NEIL1
NEIL2
NEIL3
mNeil3
(S)-Sp-10
(S)-Sp-11
B
(R)-Sp-9
(R)-Sp-10
(R)-Sp-11

Figure S3

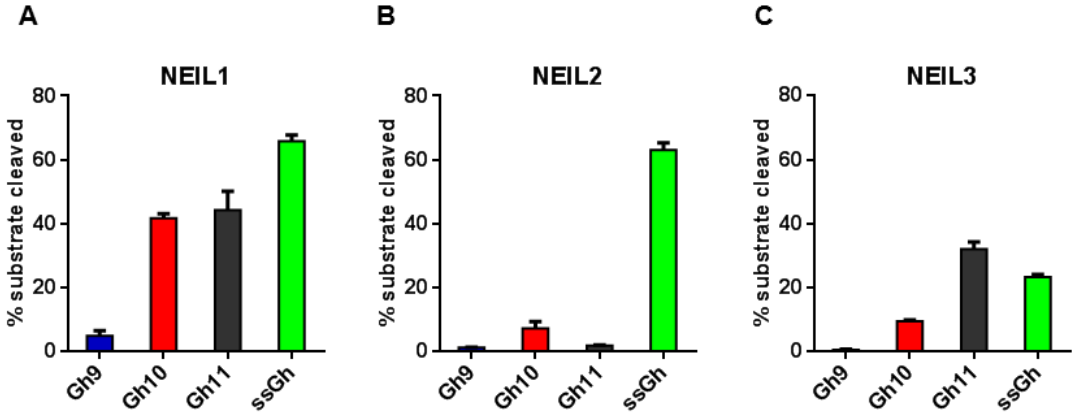
A
NEIL1
% substrate cleaved
0
20
40
60
80
Gh9
Gh10
Gh11
ssGh
B
NEIL2
% substrate cleaved
0
20
40
60
80
Gh9
Gh10
Gh11
ssGh
C
NEIL3
% substrate cleaved
0
20
40
60
80
Gh9
Gh10
Gh11
ssGh

Figure S4

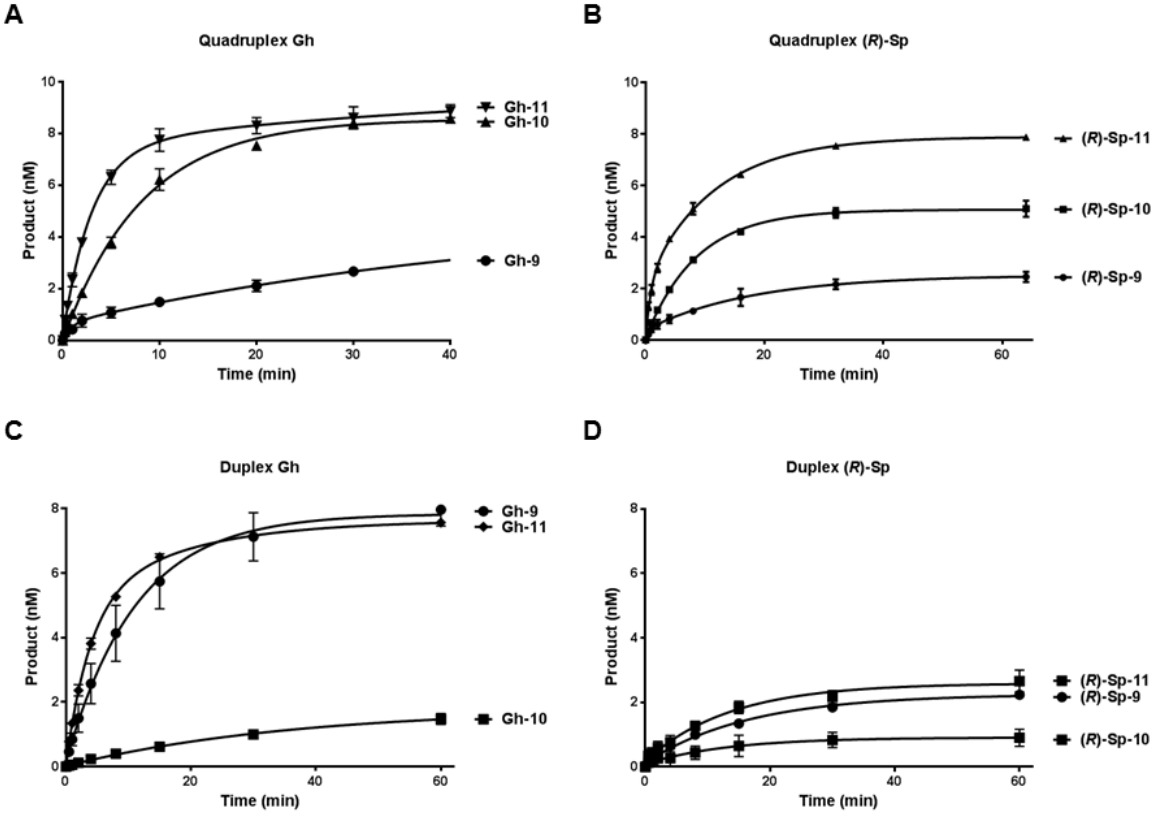
A
Quadruplex Gh
Product (nM)
Time (min)
Gh-11
Gh-10
Gh-9
B
Quadruplex (R)-Sp
Product (nM)
Time (min)
(R)-Sp-11
(R)-Sp-10
(R)-Sp-9
C
Duplex Gh
Product (nM)
Time (min)
Gh-9
Gh-11
Gh-10
D
Duplex (R)-Sp
Product (nM)
Time (min)
(R)-Sp-11
(R)-Sp-9
(R)-Sp-10

Figure S5

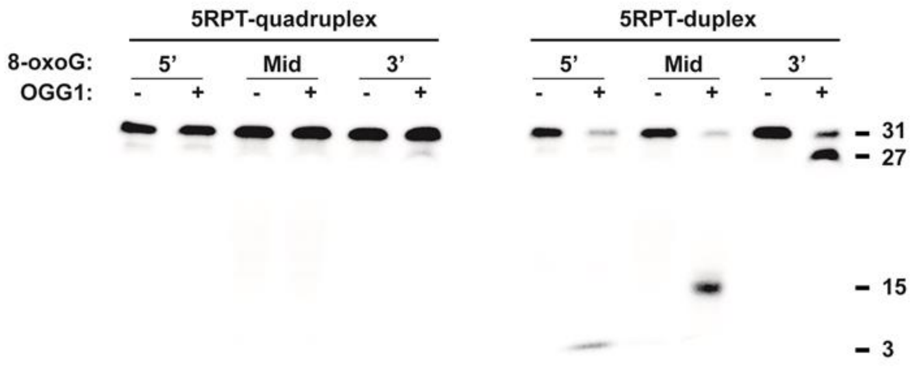
5RPT-quadruplex
5RPT-duplex
8-oxoG:
5'
Mid
3'
5'
Mid
3'
OGG1:
- + - + - + - + - + - +
31
27
15
3

Figure S6

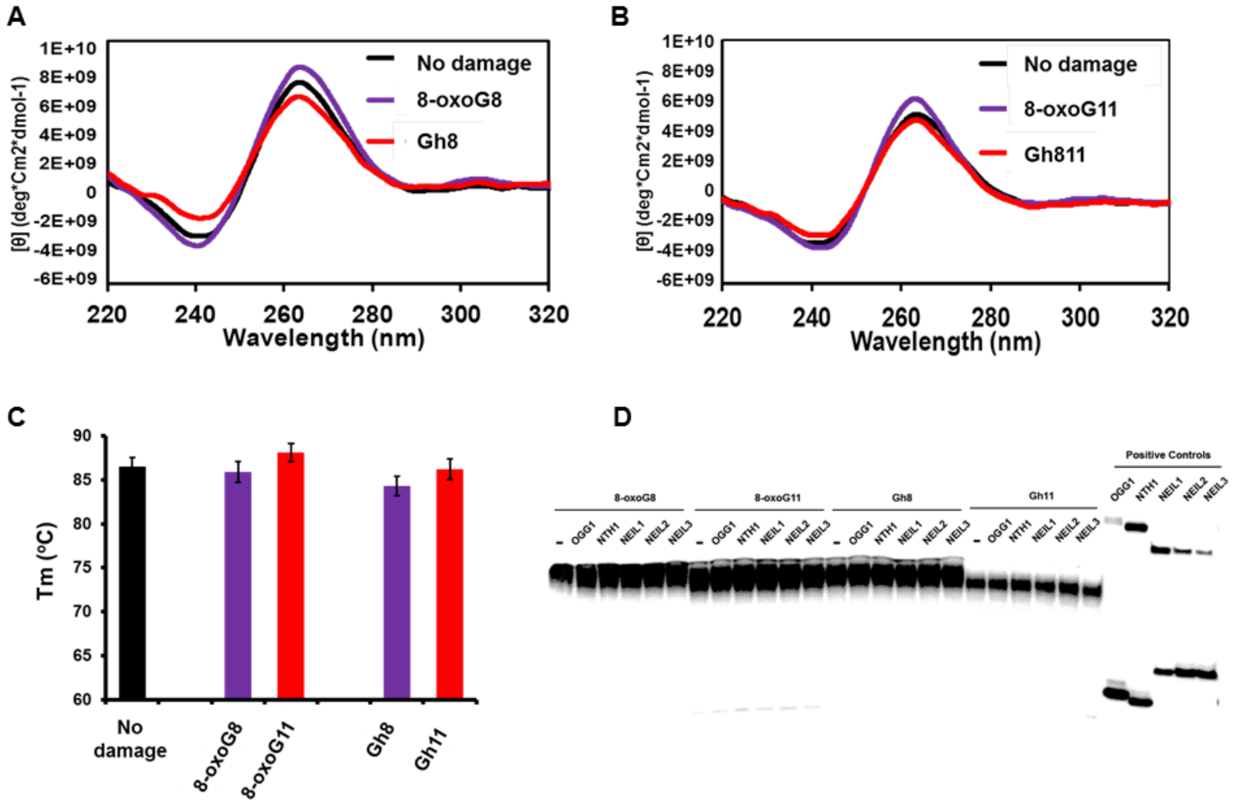
A
1E+10
8E+09
6E+09
4E+09
2E+09
0
-2E+09
-4E+09
-6E+09
[θ] (deg*Cm2*dmol-1)
No damage
8-oxoG8
Gh8
220
240
260
280
300
320
Wavelength (nm)
B
No damage
8-oxoG11
Gh811
Wavelength (nm)
C
90
85
80
75
70
65
60
Tm (°C)
No damage
8-oxoG8
8-oxoG11
Gh8
Gh11
D
8-oxoG8
8-oxoG11
Gh8
Gh11
Positive Controls
–
OGG1
NTH1
NEIL1
NEIL2
NEIL3

Figure S7

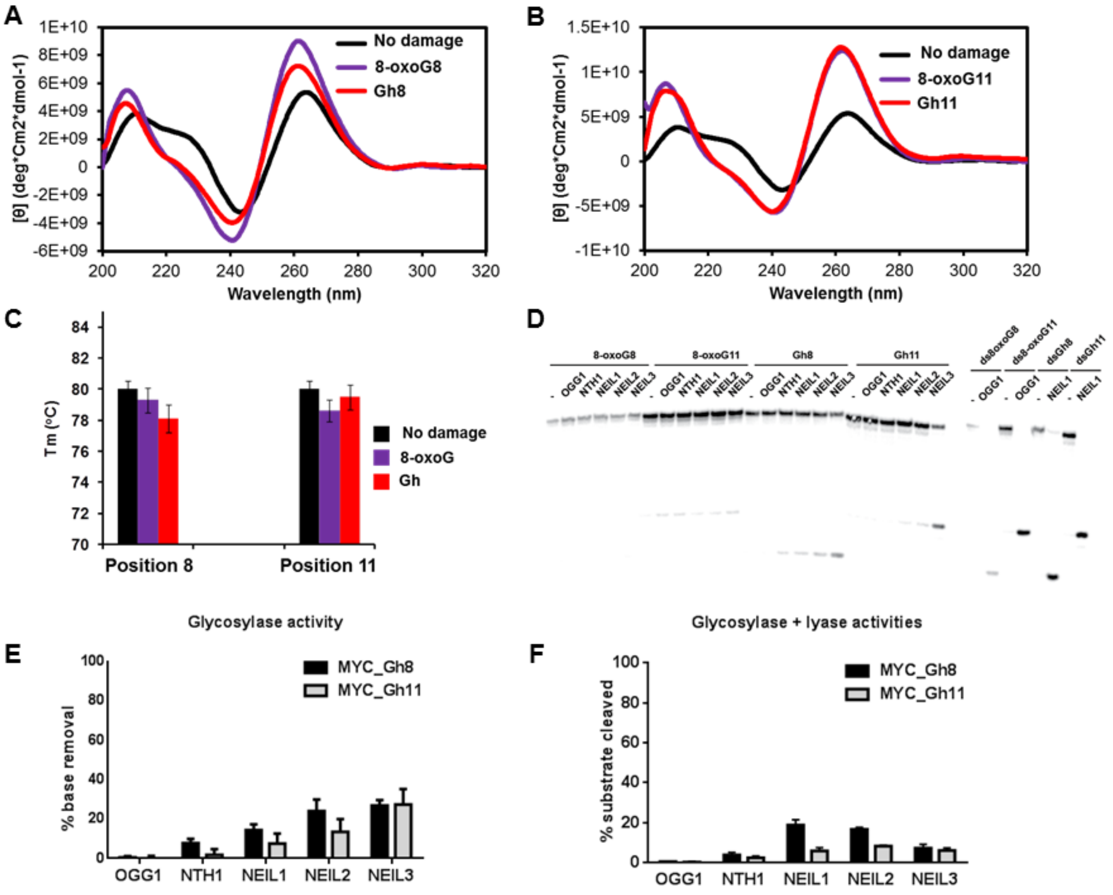

A
B
C
D
E
F
No damage
8-oxoG8
Gh8
8-oxoG11
Gh11
[θ] (deg*Cm2*dmol-1)
Wavelength (nm)
Tm (°C)
8-oxoG
Gh
Position 8
Position 11
8-oxoG8
8-oxoG11
Gh8
Gh11
OGG1
NTH1
NEIL1
NEIL2
NEIL3
Glycosylase activity
Glycosylase + lyase activities
% base removal
% substrate cleaved
MYC_Gh8
MYC_Gh11

Figure S8

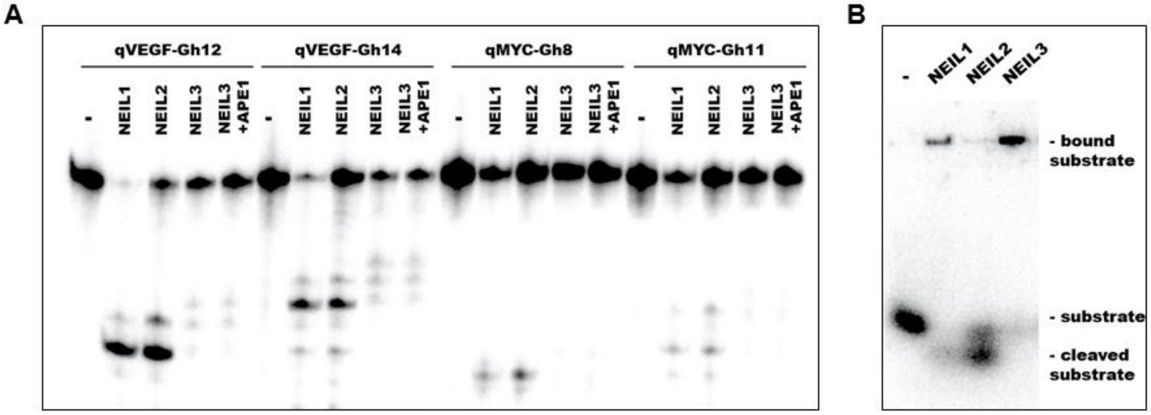
A
qVEGF-Gh12
qVEGF-Gh14
qMYC-Gh8
qMYC-Gh11
-
NEIL1
NEIL2
NEIL3
NEIL3 +APE1
B
-
NEIL1
NEIL2
NEIL3
- bound substrate
- substrate
- cleaved substrate

Figure S9

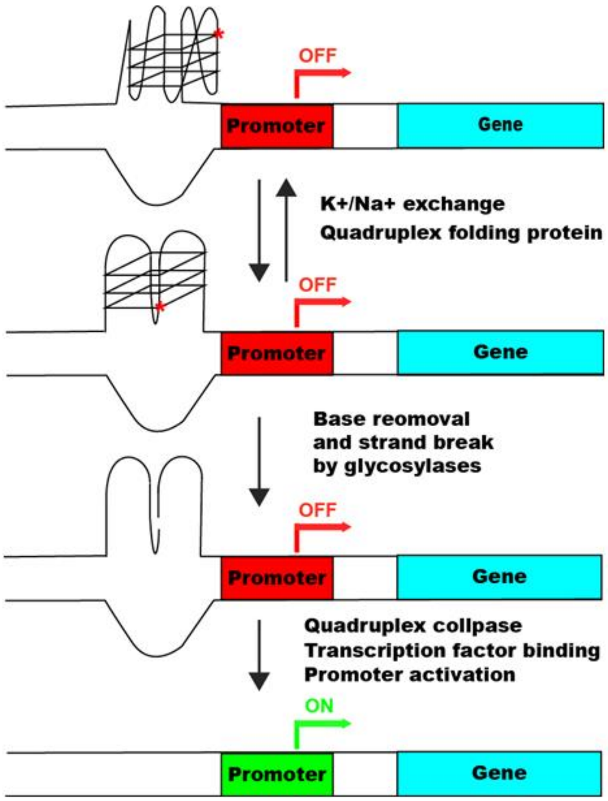

OFF
Promoter
Gene
K+/Na+ exchange
Quadruplex folding protein
OFF
Promoter
Gene
Base reomoval
and strand break
by glycosylases
OFF
Promoter
Gene
Quadruplex collpase
Transcription factor binding
Promoter activation
ON
Promoter
Gene
