# Supplementary material for: The NEIL glycosylases remove oxidized guanine lesions from telomeric and promoter quadruplex DNA structures
Source: Nucleic Acids Res. 2015 Jun 27;43(14):7171. doi: 10.1093/nar/gkv673 (PMC4538843; doi:10.1093/nar/gkv673)
Supplement: SUPPLEMENTARY DATA [file supp_43_14_7171__index.html]

The NEIL glycosylases remove oxidized guanine lesions from telomeric and promoter quadruplex DNA structures — SUPPLEMENTARY DATA 

# The NEIL glycosylases remove oxidized guanine lesions from telomeric and promoter quadruplex DNA structures

## SUPPLEMENTARY DATA

- SUPPLEMENTARY DATA
